# Supplementary material for: In-situ resonant band engineering of solution-processed semiconductors generates high performance n-type thermoelectric nano-inks
Source: Nat Commun. 2020 Apr 29;11:2069. doi: 10.1038/s41467-020-15933-2 (PMC7190739; doi:10.1038/s41467-020-15933-2)
Supplement: Supplementary file 2 — Supplementary Information [file 41467_2020_15933_MOESM2_ESM.pdf]

Supplementary Information for:

In-situ resonant band engineering of solution-processed semiconductors generates high performance n-type thermoelectric nano-inks

Ayaskanta Sahu, Boris Russ, Miao Liu, Fan Yang, Edmond W. Zaia, Madeleine P. Gordon, Jason D. Forster, Ya-Qian Zhang, Mary C. Scott, Kristin A. Persson, Nelson E. Coates, Rachel A. Segalman, and Jeffrey J. Urban*

**corresponding author, email: jjurban@lbl.gov*

Supplementary Figures

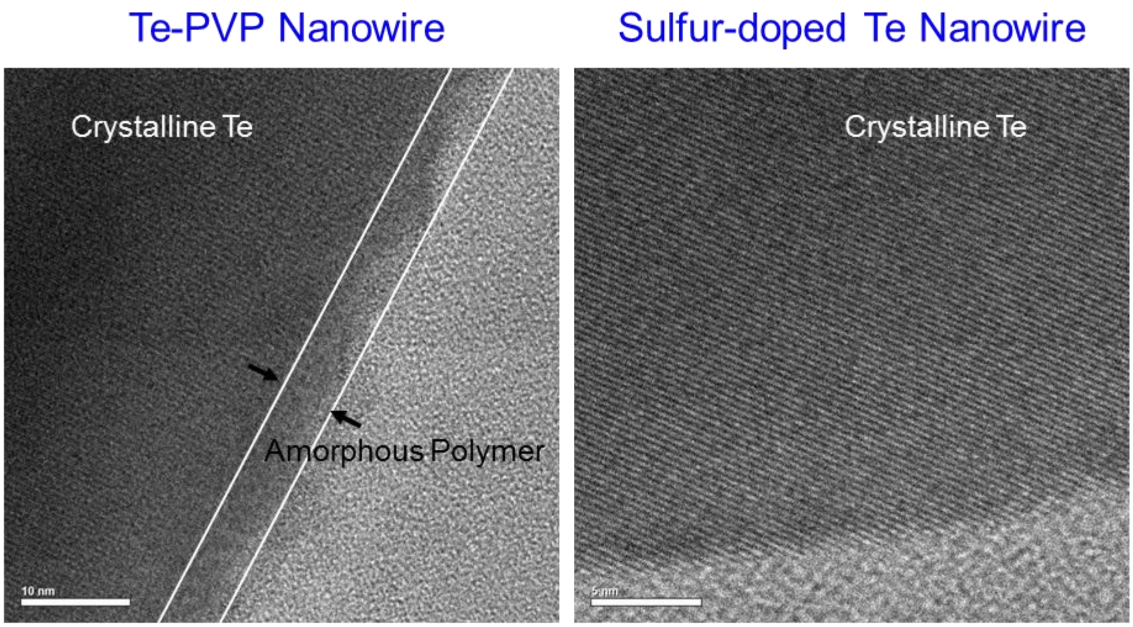

Supplementary Figure 1. Preserving crystallinity of nanowires with doping. High resolution transmission electron micrographs of undoped Te nanowires capped with polymer (polyvinylpyrrolidone, PVP) and sulfur doped Te nanowires. An amorphous polymer (PVP) layer on the surface of the tellurium nanowire with thicknesses typically on the order of 2-5 nm can be observed. In the sulfur-doped Te nanowires, the crystalline Te domain extends upto the edge of the nanowire and no amorphous layer is observed suggesting that all the polymer has been removed.

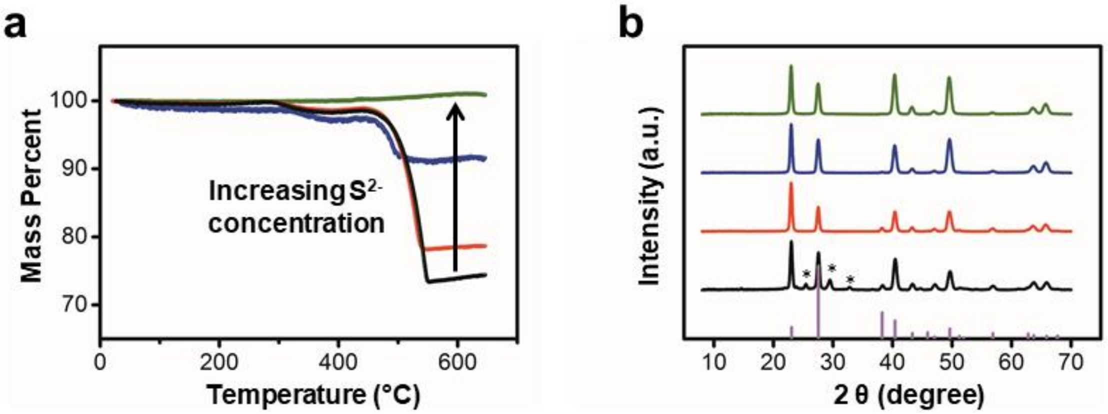

Supplementary Figure 2. Structural characterization of nanowires (a) Thermogravimetric analyses and **(b)** X-ray diffraction patterns for undoped NWs (black) and doped NWs (red – 1.2%, blue -1.5% and green -2.4% in increasing order of dopant concentration). The purple bars denote the reference peaks for pure Te while the asterisks show peaks from TeO_2. Sulfur doping prevents surface oxidation.

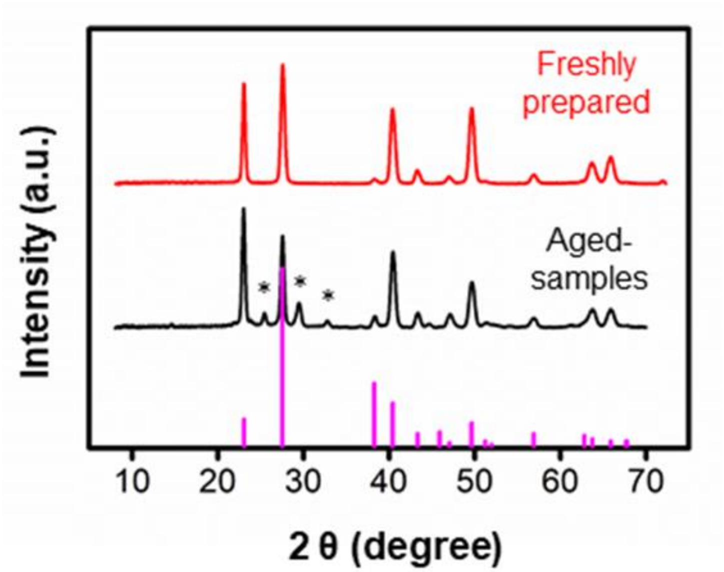

Supplementary Figure 3. Ambient stability of nanowires. X-ray diffraction patterns for undoped Te NWs that are freshly prepared (red) and stored in ambient conditions for a few weeks (red). The pink bars denote the reference peaks for pure Te while the asterisks show peaks from TeO_2.

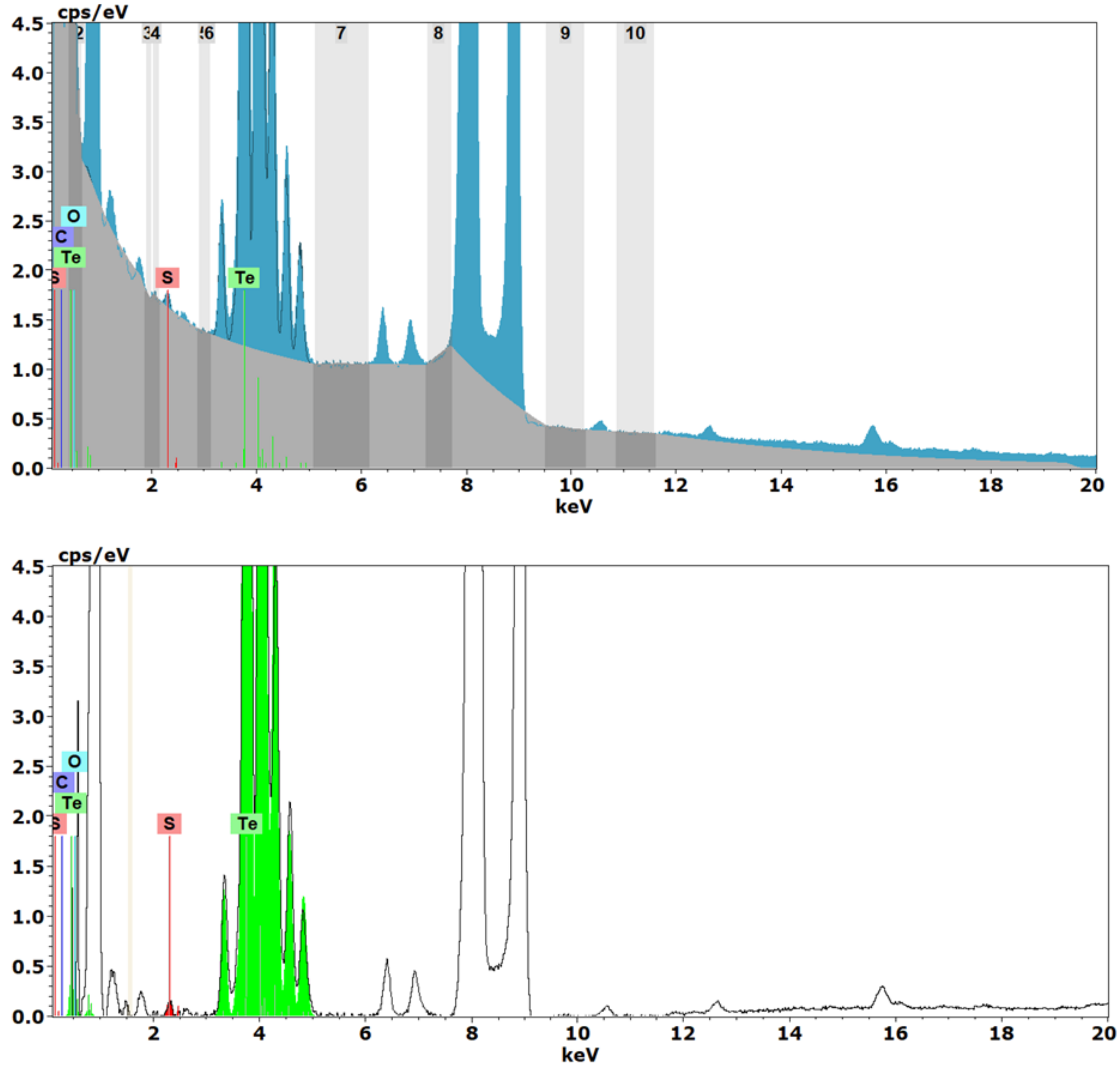

Supplementary Figure 4. STEM EDX spectrum before and after deconvolution: The spectrum shows the presence of a tiny amount of Sulfur in the S-doped Te samples. The approximate value from the spectrum is S: 0.38 at.%, Te: 99.62 at.%. However, since the S dopant concentration is too low, and the EDX data generates a systematic error around 1-2% for quantification considering the errors in background subtraction, data fitting etc., thus the absolute quantification value for S and Te are not accurate.

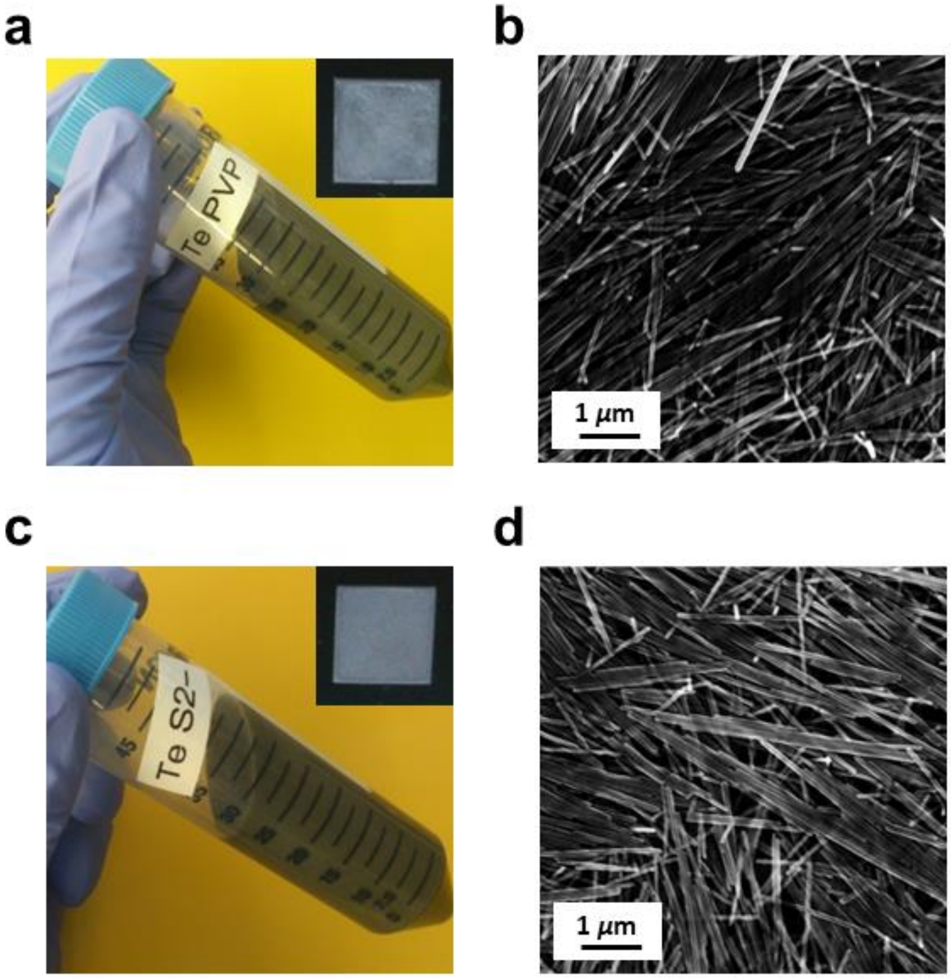

Supplementary Figure 5. Colloidal stability in water and morphology of solid thin films. Stable dispersions of **(a)** polyvinylpyrollidone-capped and **(c)** fully S^{2-}-exchanged Te NWs in water. The insets show smooth films cast from these dispersions on 1cm X 1cm square glass substrates. **(b, d)** Scanning electron micrographs of the drop-cast films in (a) and (c) respectively

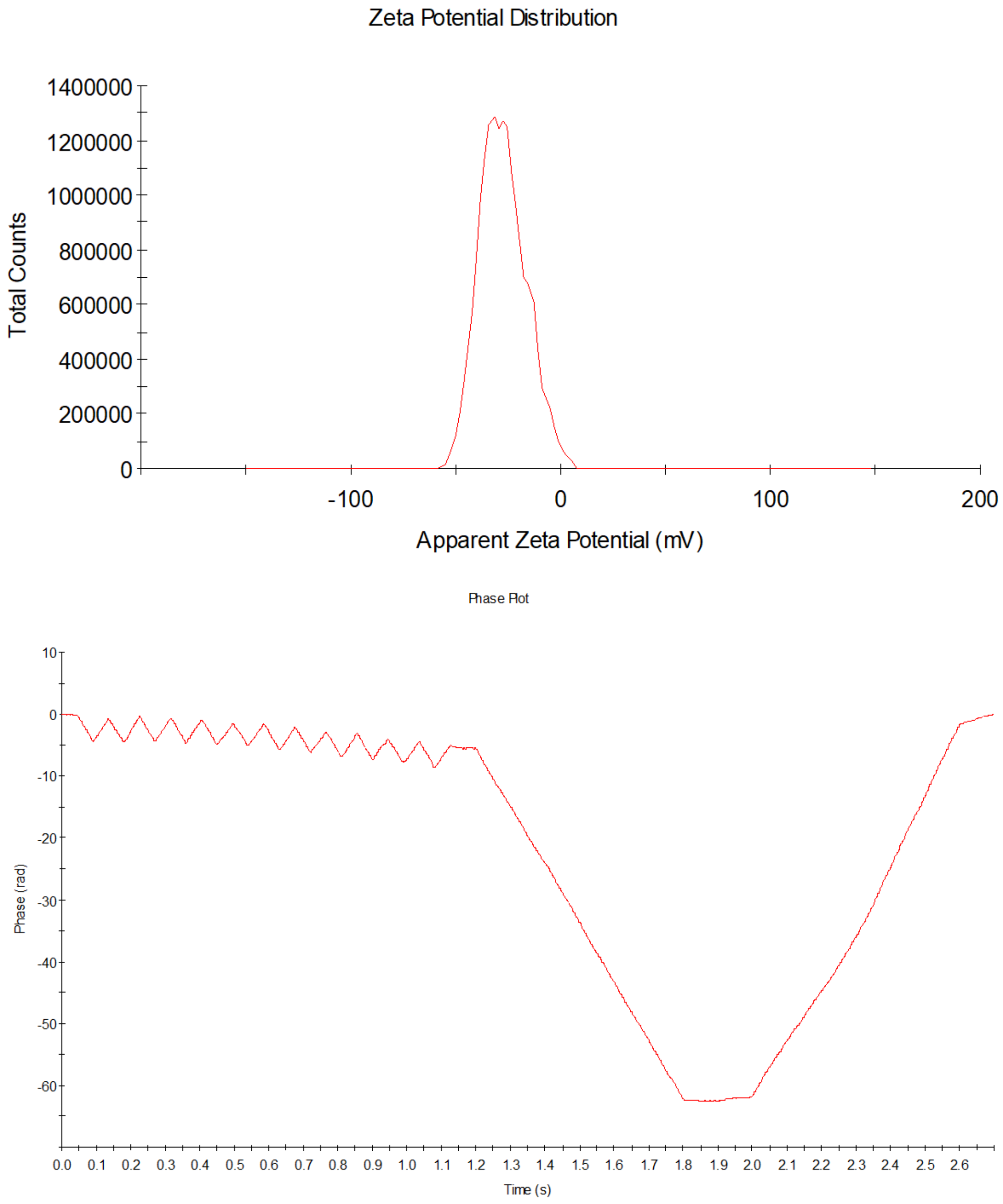

Supplementary Figure 6. Colloidal stability using Zeta Potential measurements. The averaged (n=102) Zeta Potential and phase distribution for sulfur capped tellurium nanowires (~2.4% sulfur concentration) dispersed in water. Sample concentration was ~10 microgram/mL. The sample was transferred to a zeta cell (Malvern Instruments) and measured at 25 °C. The zeta potential was -27.5 ± -10.5 mV (n=102). A viscosity of 0.8872 cP, a dielectric constant of 78.5 and Henry function of 1.5 were used for the calculations.

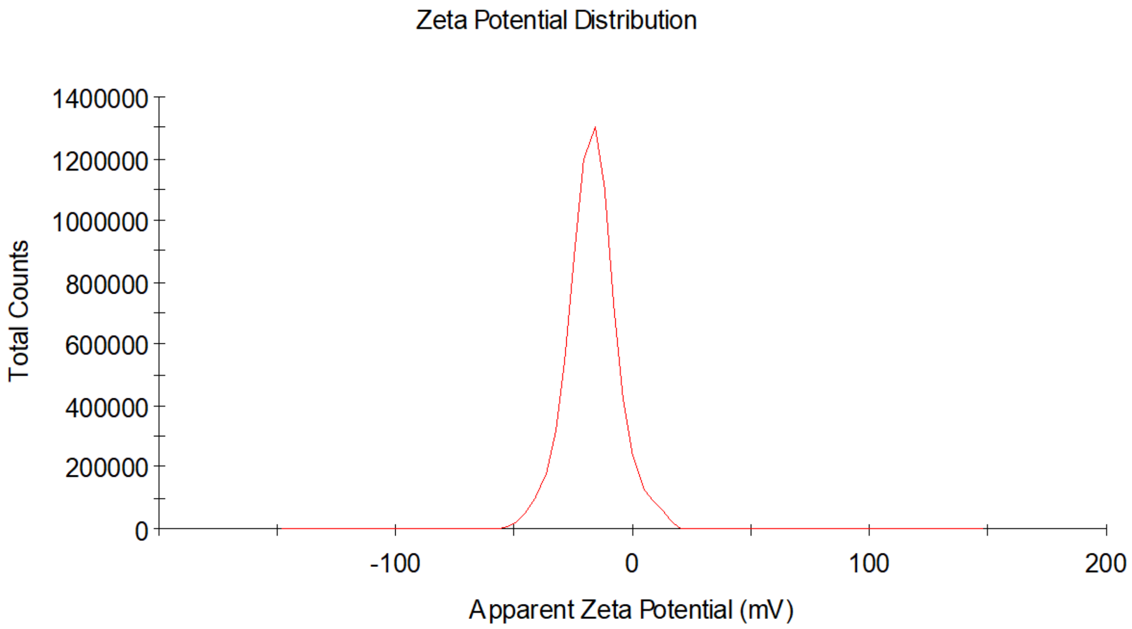

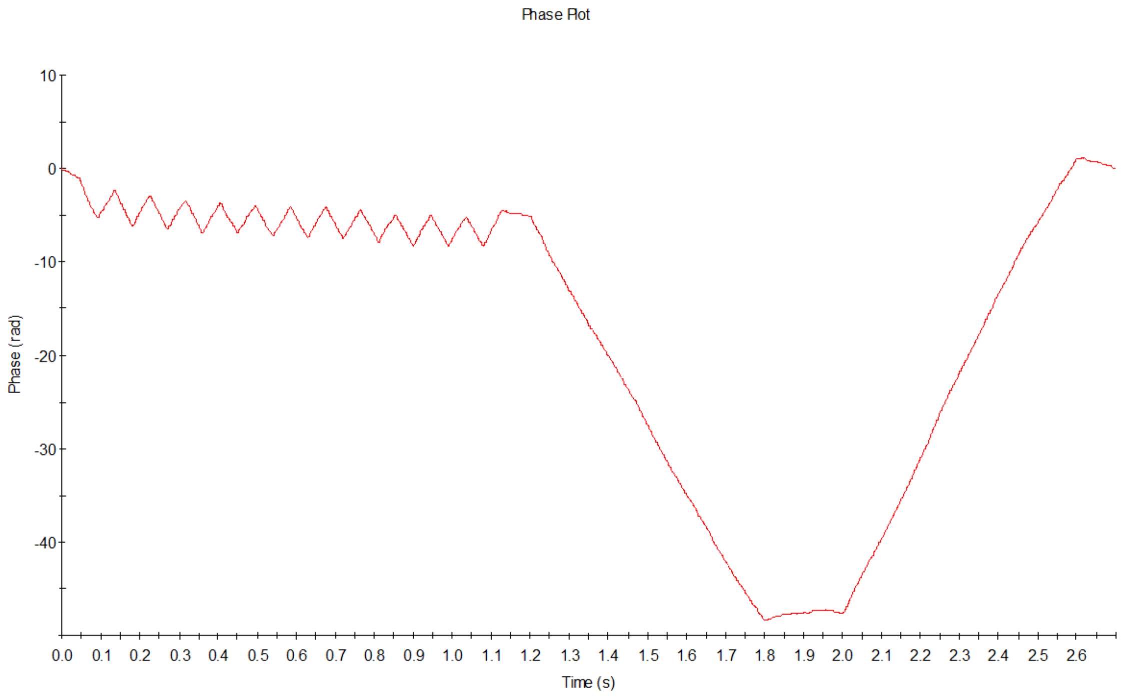

Supplementary Figure 7. Colloidal stability using Zeta Potential measurements. The averaged (n=72) Zeta Potential and phase distribution for sulfur capped tellurium nanowires (~2.2% sulfur concentration) dispersed in water. Sample concentration was ~10 microgram/mL. The sample was transferred to a zeta cell (Malvern Instruments) and measured at 25 °C. The zeta potential was -18.1 $\pm$ -10.1 mV (n=72). A negative zeta potential value suggests that the surface is negatively charged. A viscosity of 0.8872 cP, a dielectric constant of 78.5 and Henry function of 1.5 were used for the calculations.

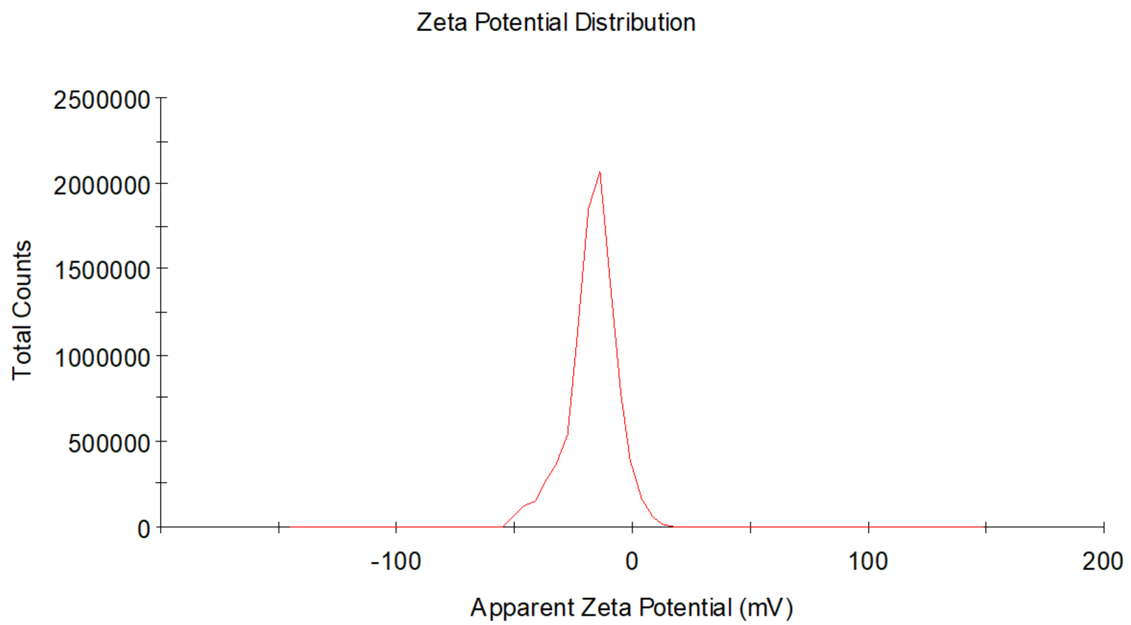

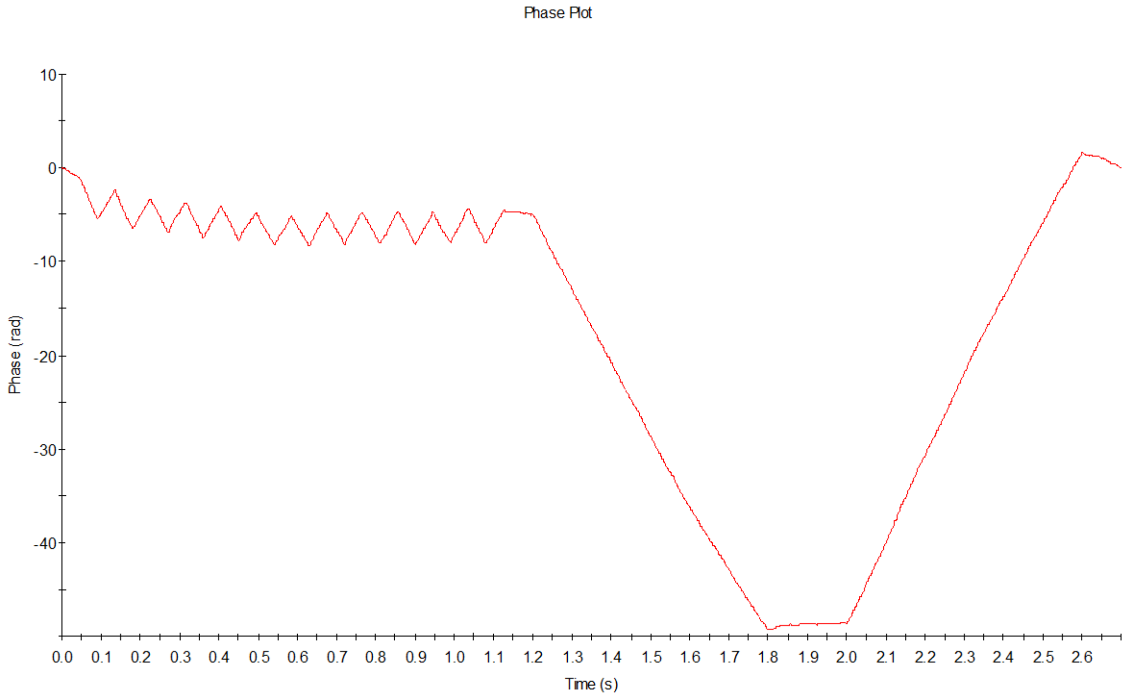

Supplementary Figure 8. Colloidal stability using Zeta Potential measurements. The averaged (n=72) Zeta Potential and phase distribution for sulfur capped tellurium nanowires (~1.8% sulfur concentration) dispersed in water. Sample concentration was ~10 microgram/mL. The sample was transferred to a zeta cell (Malvern Instruments) and measured at 25 °C. The zeta potential was -16.8 ± -10.2 mV (n=72). A negative zeta potential value suggests that the surface is negatively charged. A viscosity of 0.8872 cP, a dielectric constant of 78.5 and Henry function of 1.5 were used for the calculations.

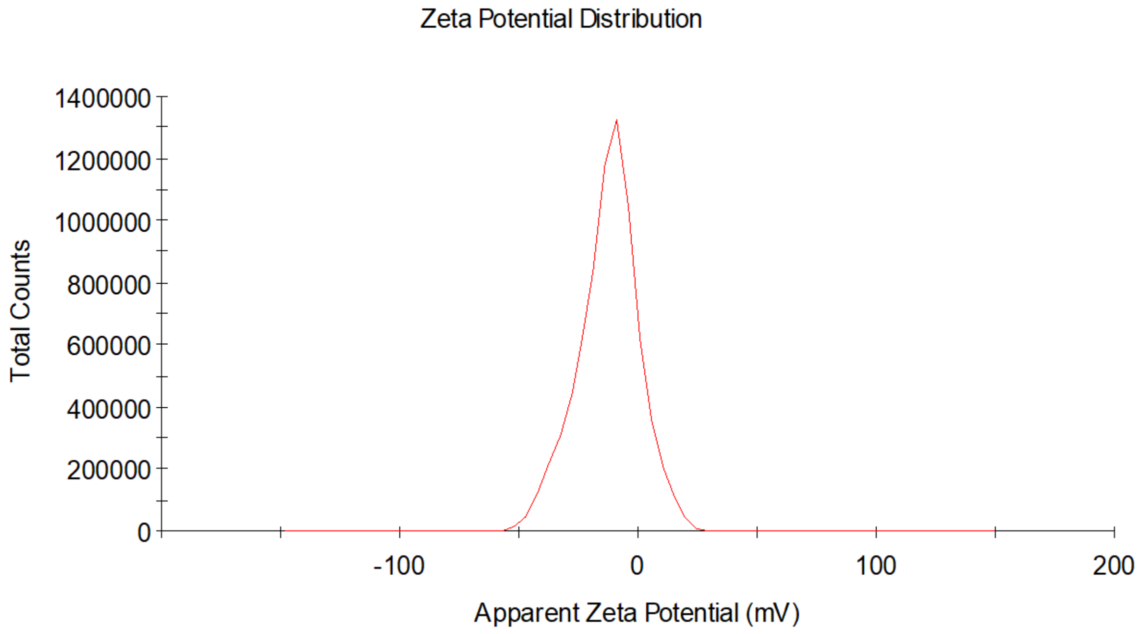

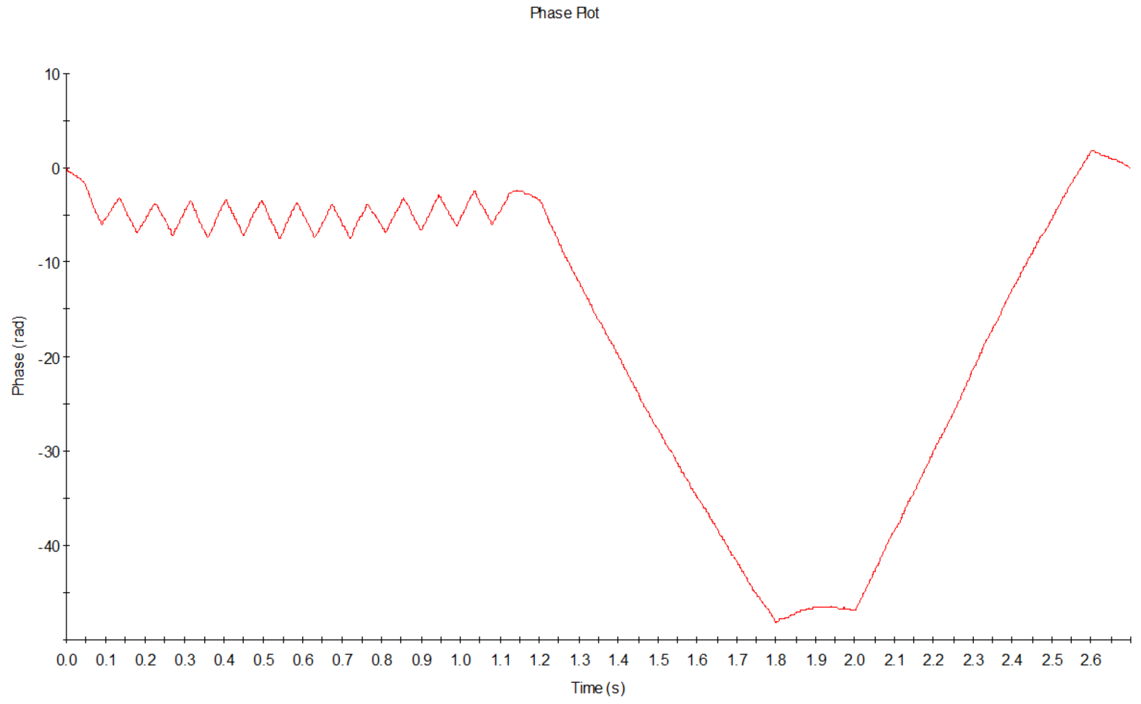

Supplementary Figure 9. Colloidal stability using Zeta Potential measurements. The averaged (n=72) Zeta Potential and phase distribution for sulfur capped tellurium nanowires (~1.5% sulfur concentration) dispersed in water. Sample concentration was ~10 microgram/mL. The sample was transferred to a zeta cell (Malvern Instruments) and measured at 25 °C. The zeta potential was -12.3 ± -12.8 mV (n=72). A negative zeta potential value suggests that the surface is negatively charged. A viscosity of 0.8872 cP, a dielectric constant of 78.5 and Henry function of 1.5 were used for the calculations.

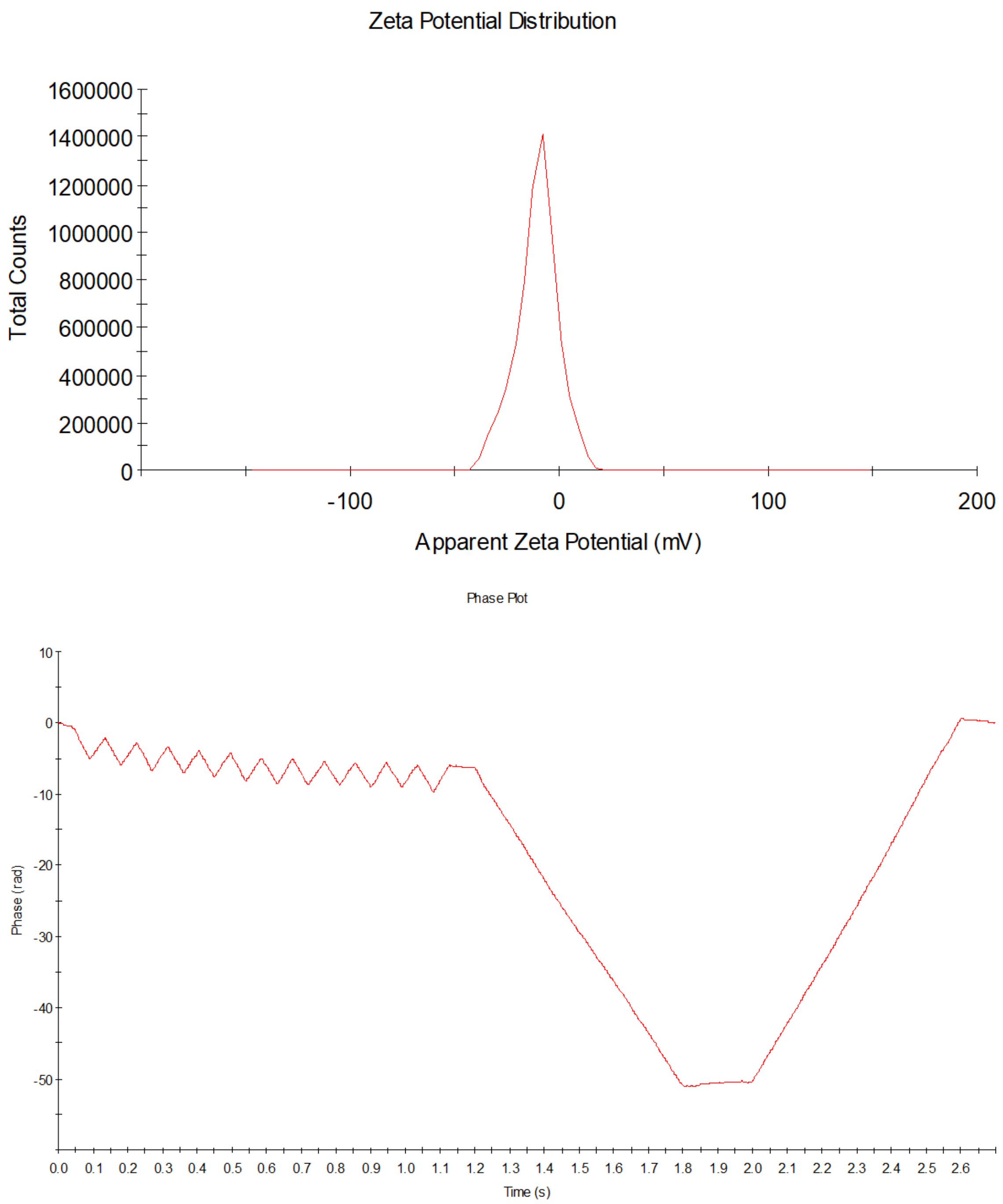

Supplementary Figure 10. Colloidal stability using Zeta Potential measurements. The averaged (n=72) Zeta Potential and phase distribution for sulfur capped tellurium nanowires (~1.3% sulfur concentration) dispersed in water. Sample concentration was ~10 microgram/mL. The sample was transferred to a zeta cell (Malvern Instruments) and measured at 25 °C. The zeta potential was -10.6 ± -10.1 mV (n=72). A negative zeta potential value suggests that the surface is negatively charged. A viscosity of 0.8872 cP, a dielectric constant of 78.5 and Henry function of 1.5 were used for the calculations.

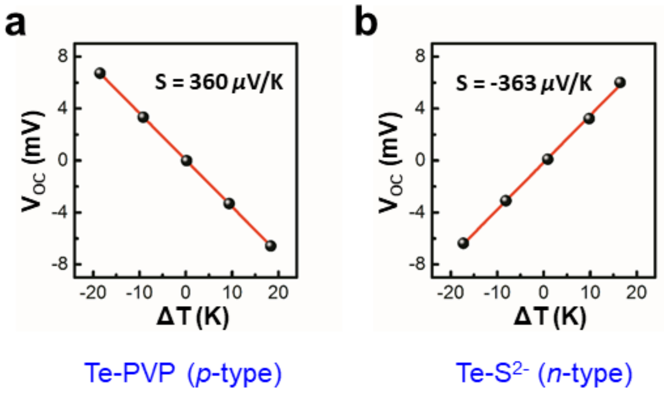

Supplementary Figure 11. Thermopower measurements on thin films of nanowires. Open circuit voltage versus applied temperature gradient for (**a**) polyvinylpyrollidone-capped (Te-PVP) and **(b)** fully S^{2-}-exchanged Te NW films (Te-S^{2-}). The Seebeck coefficient is derived from the slope of the linear fit (R^2 values of 0.9999 and 0.9996 respectively). Error bars representing the standard deviation from averaging 10 readings for each temperature gradient are captured within the data marker.

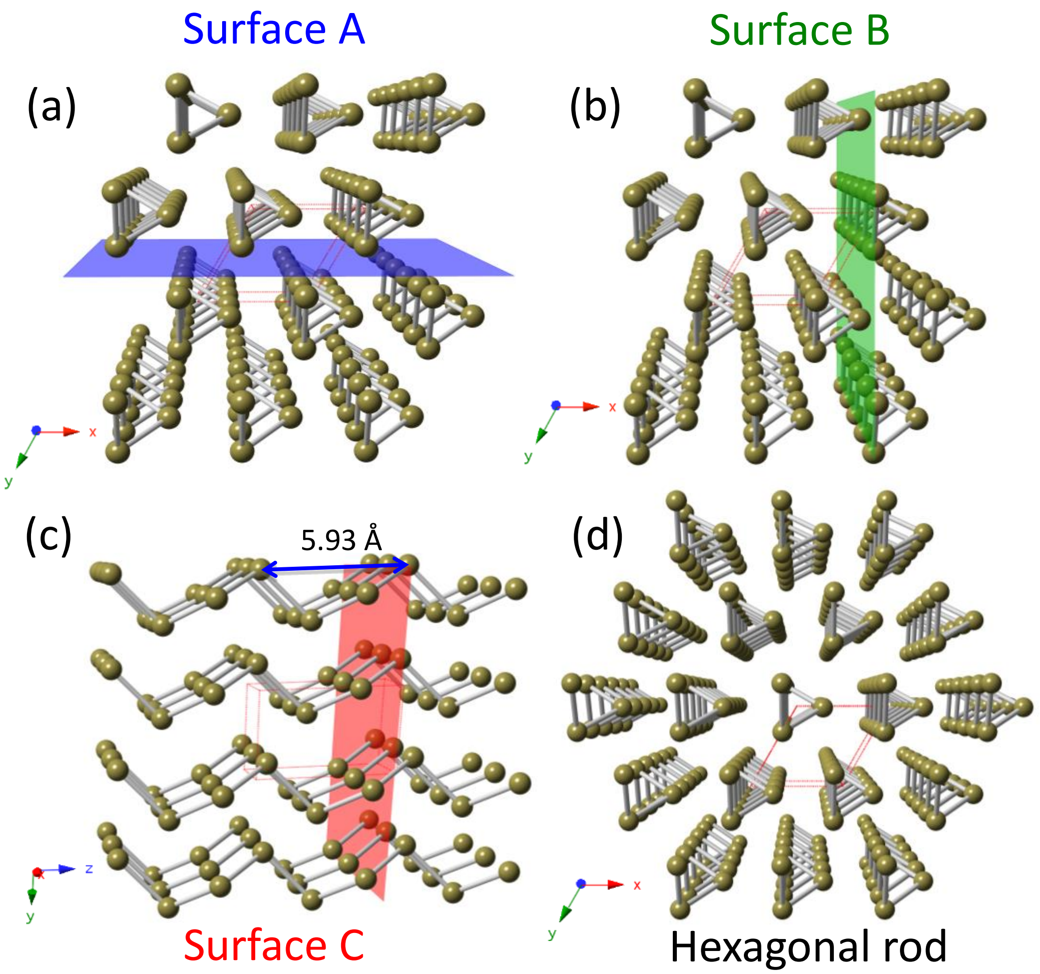

Supplementary Figure 12. **Illustrations of surfaces and atomistic structure nanorod .** (a), (b) and (c) subplots represent (010), (210) and (001) surfaces, which are denoted as surfaces A, B and C, respectively. The unit cell of tellurium crystal is shown as the tetragonal red dashed box in each plot. As exhibited by (c) and (d), the nanorod grows along the (001) direction with a 5.93Å spacing between two lattice planes. The most stable surface A drives the nanorod to form the hexagonal shape.

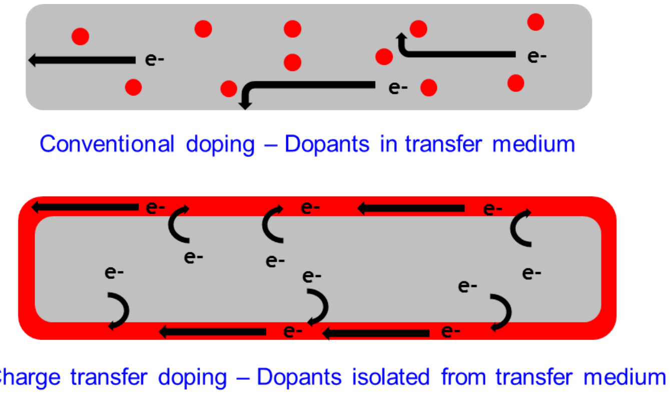

Supplementary Figure 13. Charge Transfer Doping vs Conventional Impurity Doping. At extremely high dopant concentrations typically employed for thermoelectric materials, reduced carrier scattering via charge transfer/modulation doping could lead to higher mobility charge carriers. We employ this approach at the single nanowire level.

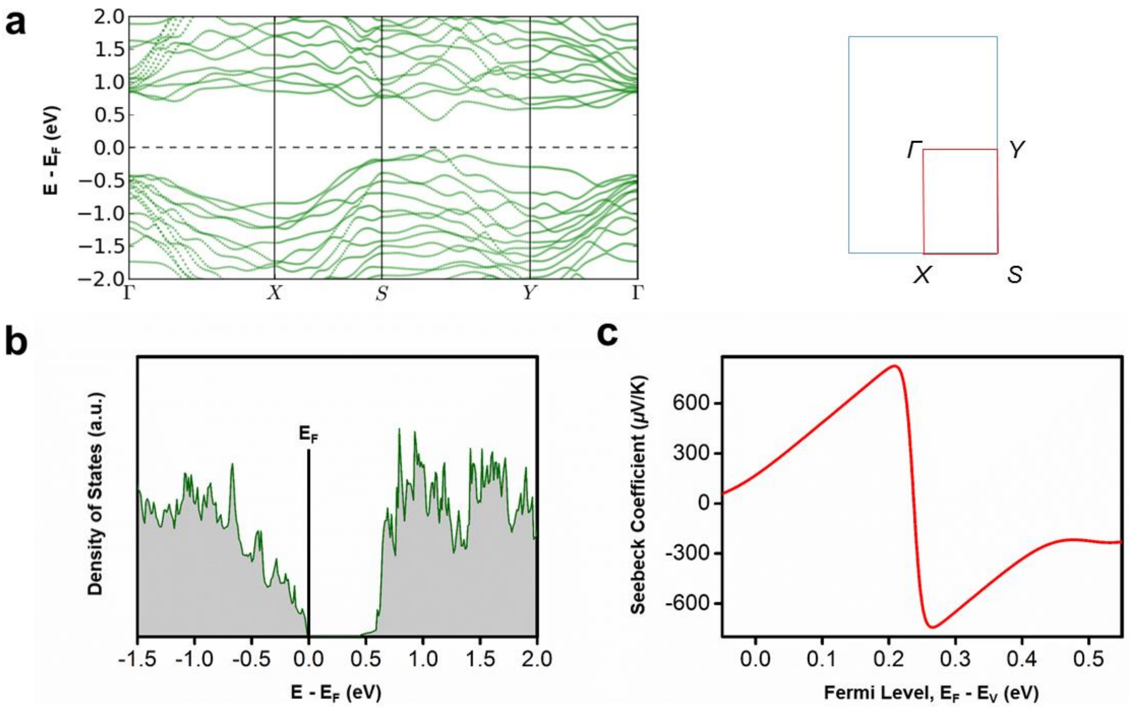

Supplementary Figure 14. DFT calculations for tellurium. (a) Band structure of tellurium obtained with GGA as implemented in the PAW scheme using VASP **(b)** Calculated density of states (DOS) **(c)** Model depicting the variation in Seebeck coefficient as a function of the Fermi level (E_F) in Te, taking into account both conduction (with effective mass $m_e^* = 0.06m_0$, where m_0 is the mass of free electron) and valence bands ($m_e^* = 0.114m_0$). E_V denotes the valence band edge.

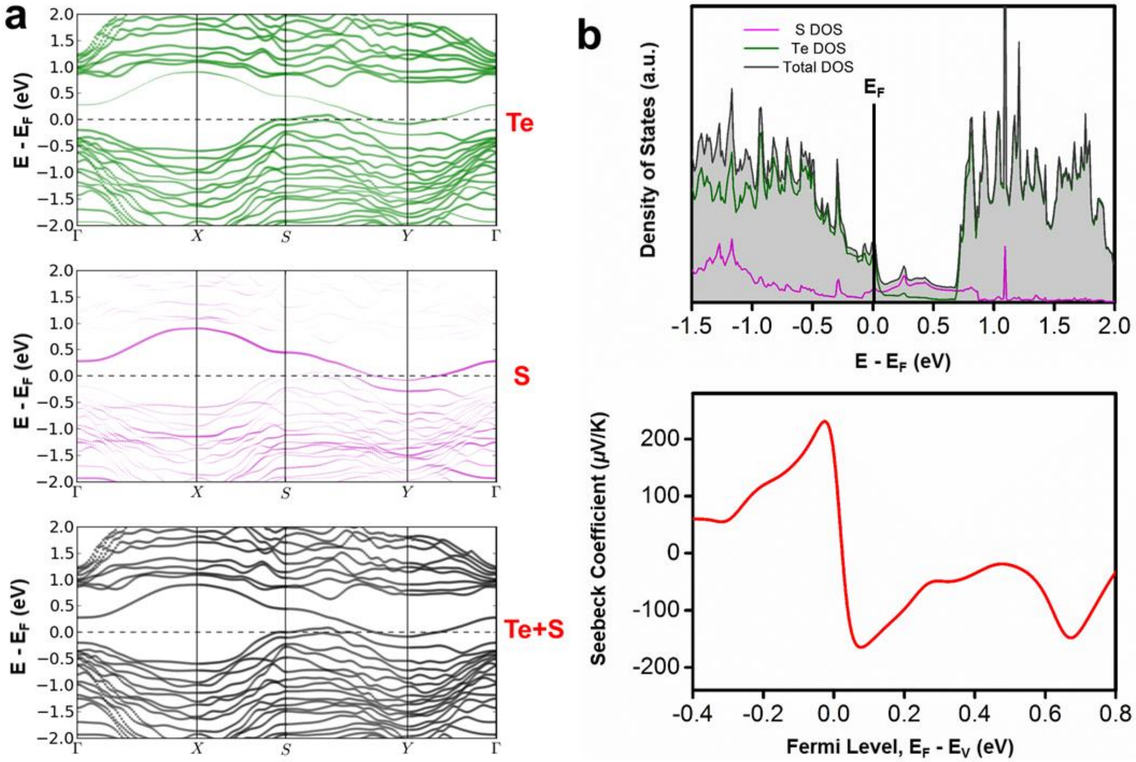

Supplementary Figure 15. DFT calculations for physisorbed sulfur on tellurium. (a) Band structure of sulfur-doped tellurium where the sulfur is physically adsorbed on the surface of tellurium obtained with GGA as implemented in the PAW scheme using VASP **(b)** Calculated density of states (DOS) **(c)** Model depicting the variation in Seebeck coefficient as a function of the Fermi level (E_F) taking into account both conduction (with effective mass $m_e^* = 0.06m_0$, where m_0 is the mass of free electron) and valence bands ($m_e^* = 0.114m_0$). E_V denotes the valence band edge.

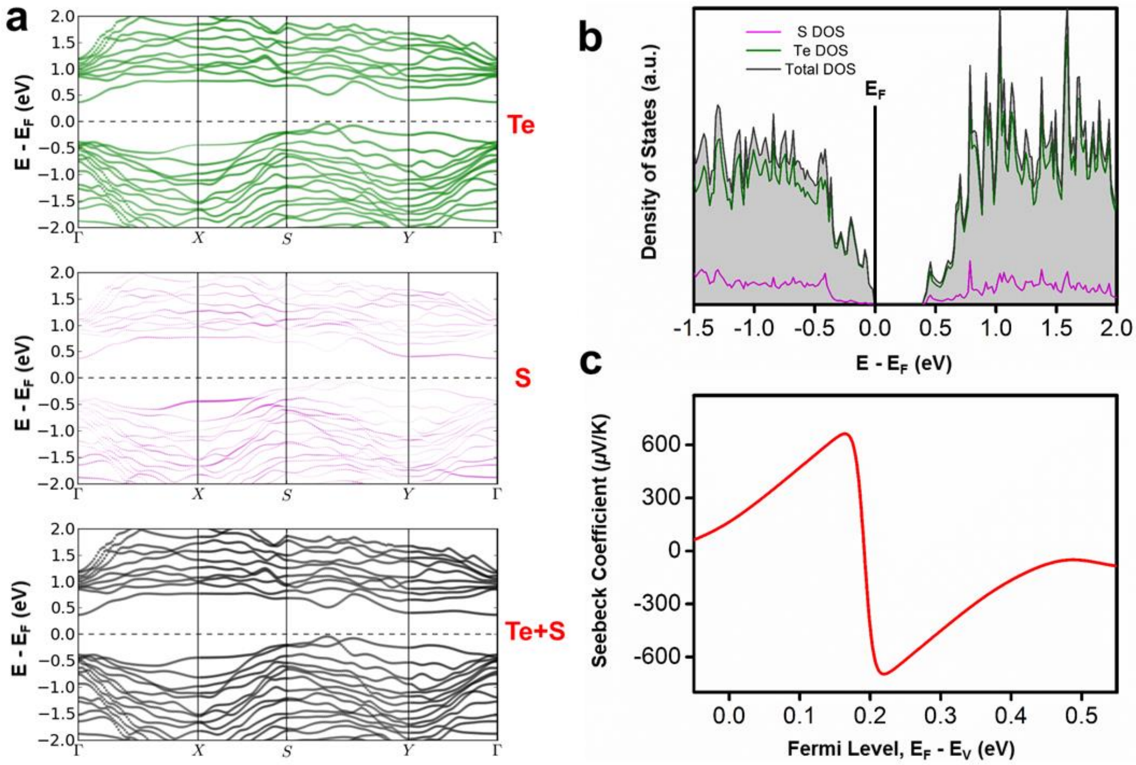

Supplementary Figure 16. DFT calculations for chemisorbed sulfur on tellurium. (a) Band structure of sulfur-doped tellurium where the sulfur is chemically adsorbed on the surface of tellurium obtained with GGA as implemented in the PAW scheme using VASP **(b)** Calculated density of states (DOS) **(c)** Model depicting the variation in Seebeck coefficient as a function of the Fermi level (E_F) taking into account both conduction (with effective mass $m_e^* = 0.06m_0$, where m_0 is the mass of free electron) and valence bands ($m_e^* = 0.114m_0$). E_V denotes the valence band edge.

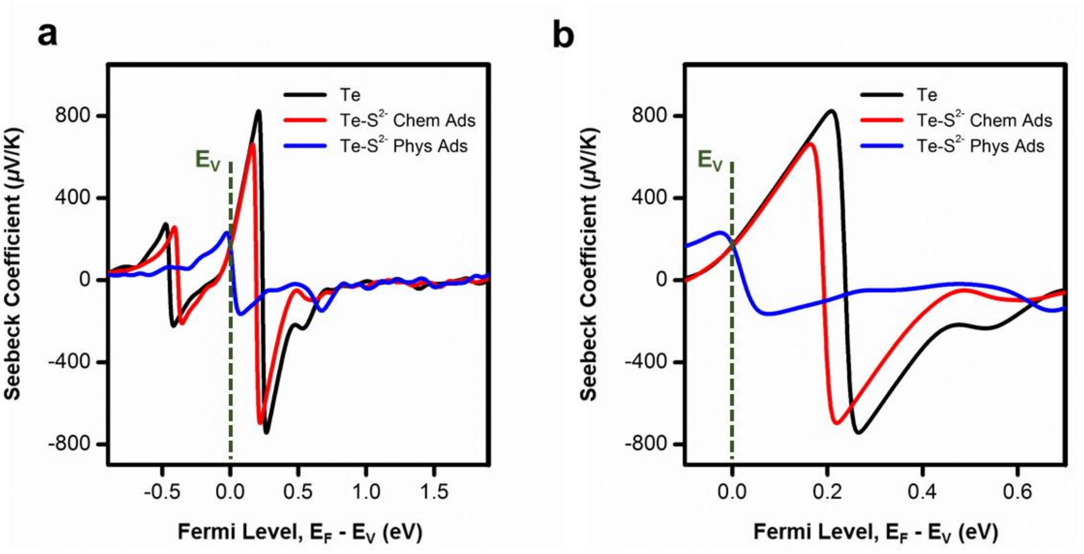

Supplementary Figure 17. Computed values of Seebeck coefficient from predicted band structures. (a) Model depicting the variation in Seebeck coefficient as a function of the Fermi level (E_F) taking into account both conduction (with effective mass $m_e^* = 0.06m_0$, where m_0 is the mass of free electron) and valence bands ($m_e^* = 0.114m_0$). E_V denotes the valence band edge. Comparison between undoped tellurium and doped tellurium with chemically adsorbed and physically adsorbed sulfur. **(b)** Zoomed-in figure of (a) focusing on the region in around the valence band edge.

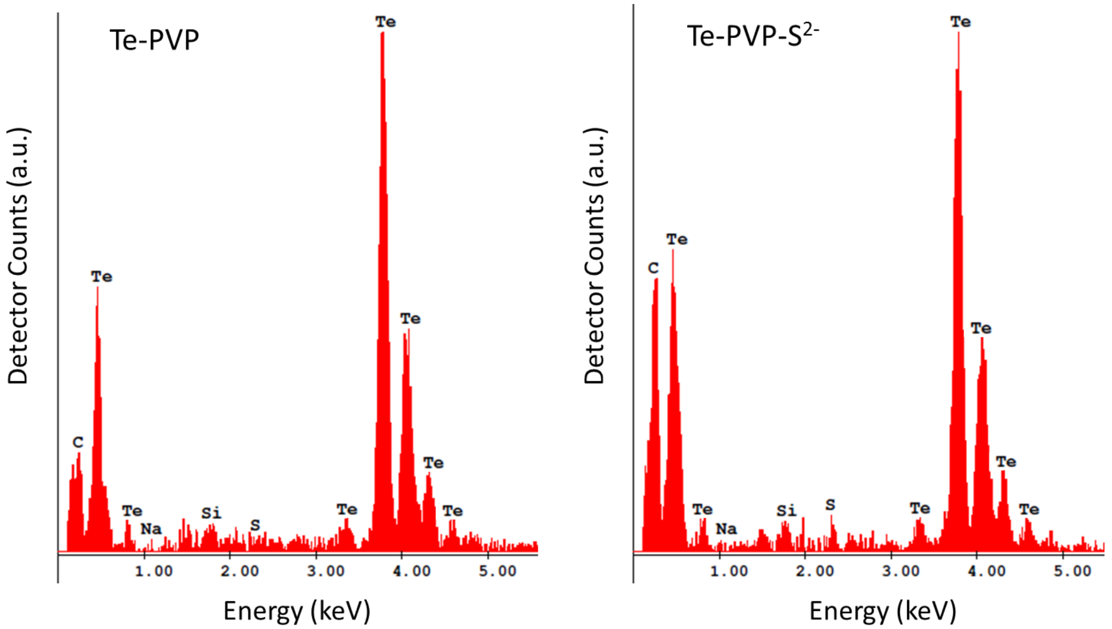

Supplementary Figure 18. **Energy Dispersive X-ray Analyses**. EDX spectra acquired during SEM imaging indicates presence of both Tellurium from the nanowires and Sulfur on the surface. A weak almost negligible sulfur peak in the undoped Te nanowires (Te-PVP) could be due to sulfur impurities from the substrate or the sample while a much stronger peak is observed in the doped nanowires (Te-PVP-S^{2-}). The Si peak is due to the substrate. The lack of an identifiable Na peak indicates effective removal of Na_2S or unbound S^{2-} ions.

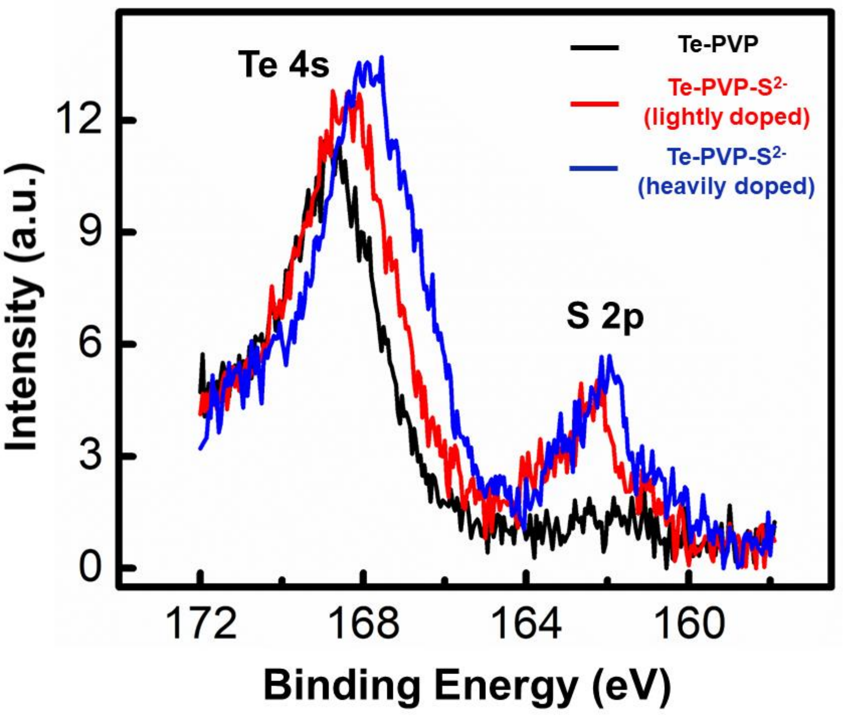

Supplementary Figure 19. **X-ray photoelectron spectra – sulfur edge**. High-resolution normalized XPS spectra of undoped Te nanowires (Te-PVP) in black and lightly and heavily doped nanowires (Te-PVP-S^{2-}) in red and blue respectively. In the Te-PVP nanowires there is no peak in the spectrum around 162-162.5 eV which suggests the lack of sulfide species. The presence of a peak around 169-170 eV in undoped Te nanowires corresponds to the Te 4s peak. The apparent red shifting of the peak at 169 eV with increased doping is due to presence of oxidized sulfur species such as sulfates and sulfites which demonstrate S 2p peaks around 169 eV and 166.5 eV respectively leading to a convolution of the Te 4s and S 2p peaks.

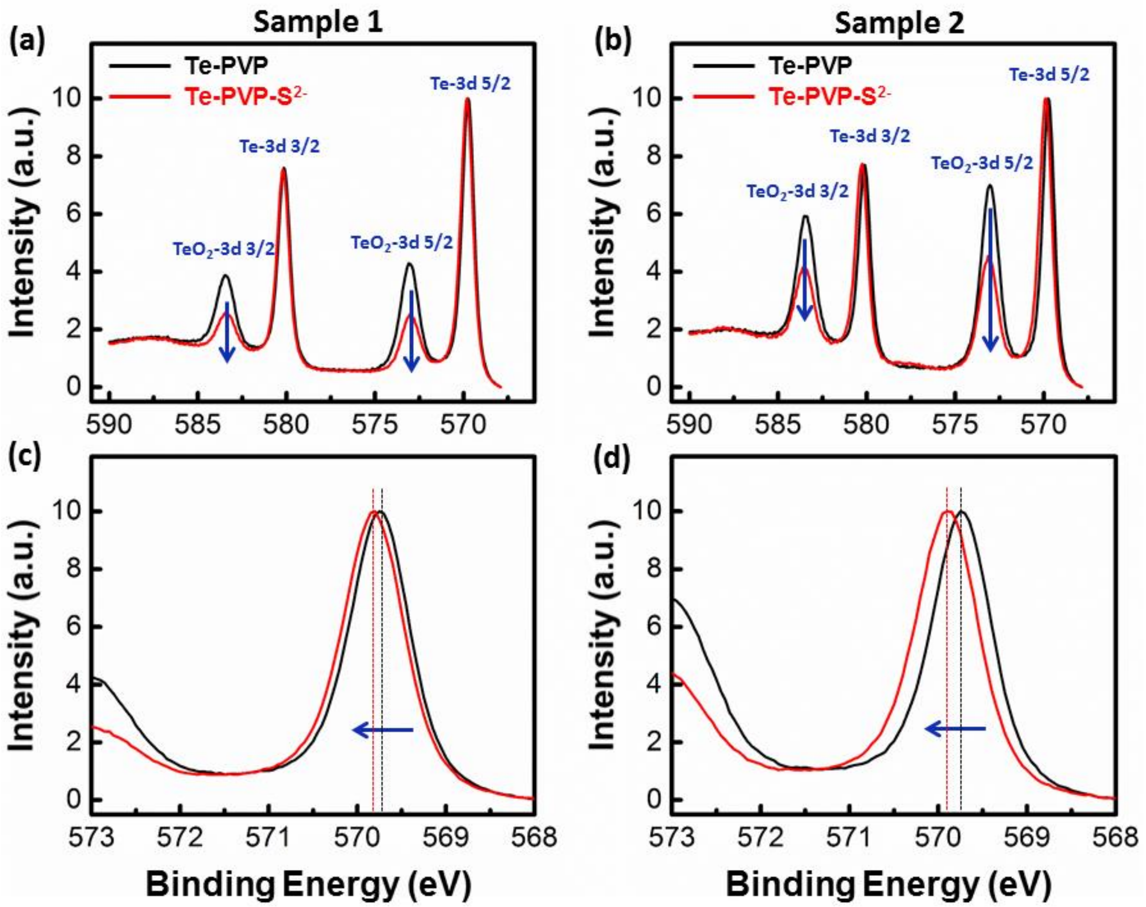

Supplementary Figure 20. **X-ray photoelectron spectra – tellurium edge (a, b)** High-resolution XPS spectra of the Te-3d peak of undoped Te nanowires (Te-PVP) in black and doped nanowires (Te-PVP-S^{2-}) in red for two different samples. In both samples, the TeO_2 peak is suppressed with doping proving that S^{2-} dopants passivate the nanowire surface effectively thus reducing oxidation. A slight blue shift to higher energies or an increase in binding energies can be observed in the zoomed-in images **(c, d)** for the doped samples which is suggestive of *p*-type doping.

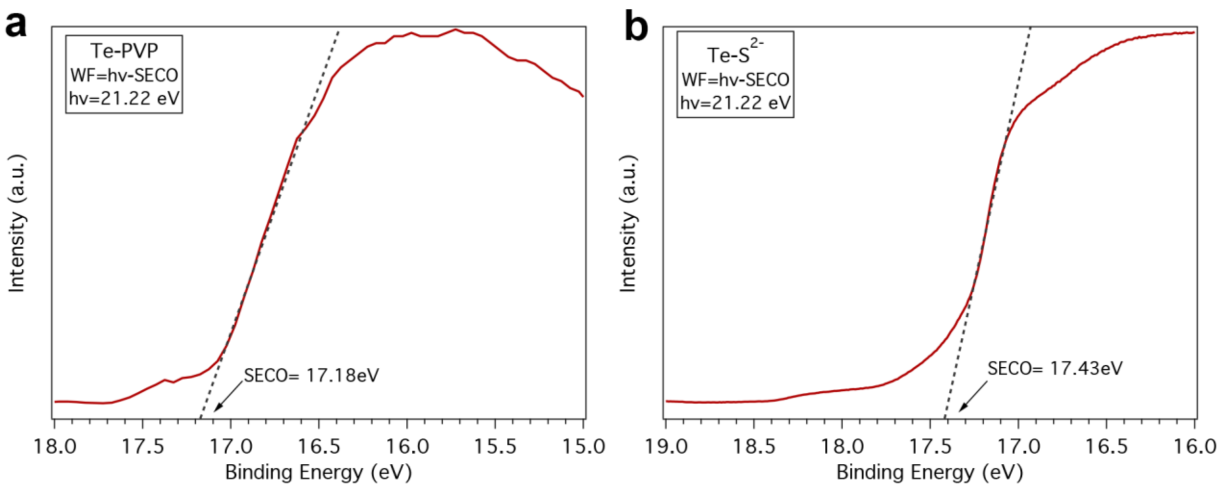

Supplementary Figure 21. Ultaviolet photoelectron spectroscopy to determine Fermi level. Representative UPS spectra of a set of (a) Te-PVP nanowires and (b) Te-S^{2-} nanowires The SECO regions are shown in the figures and are used to extract work function (WF) values of 4.04 eV and 3.8 eV for the Te-PVP and the Te-S^{2-} nanowire samples respectively. Please note that the WF values of 4 eV and 3.7 eV reported in the main text are an average from at least 3 measurements on each type of sample.

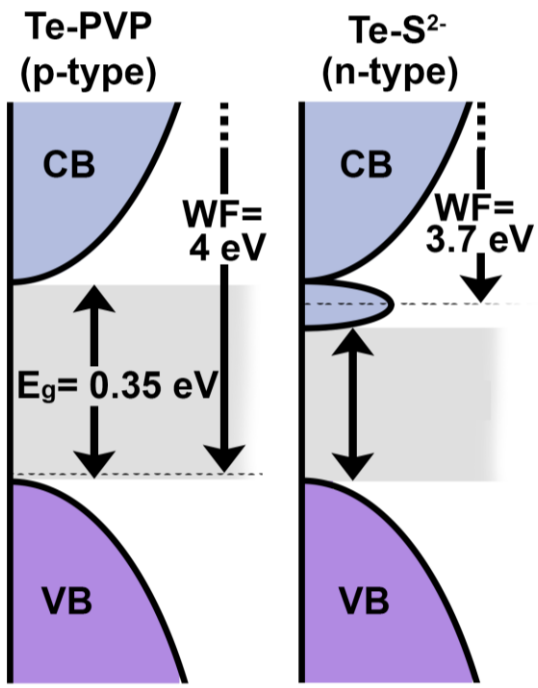

Supplementary Figure 22. Cartoon demonstrating the shift in work function due to sulfur doping. (work function values are taken from Table 1 in main text). With a band gap of 0.35 eV for bulk Te, the change in work function by 0.3 eV implies that the Fermi level of the S-doped Te NWs lies either in the dopant band or very close to the conduction band of Te.

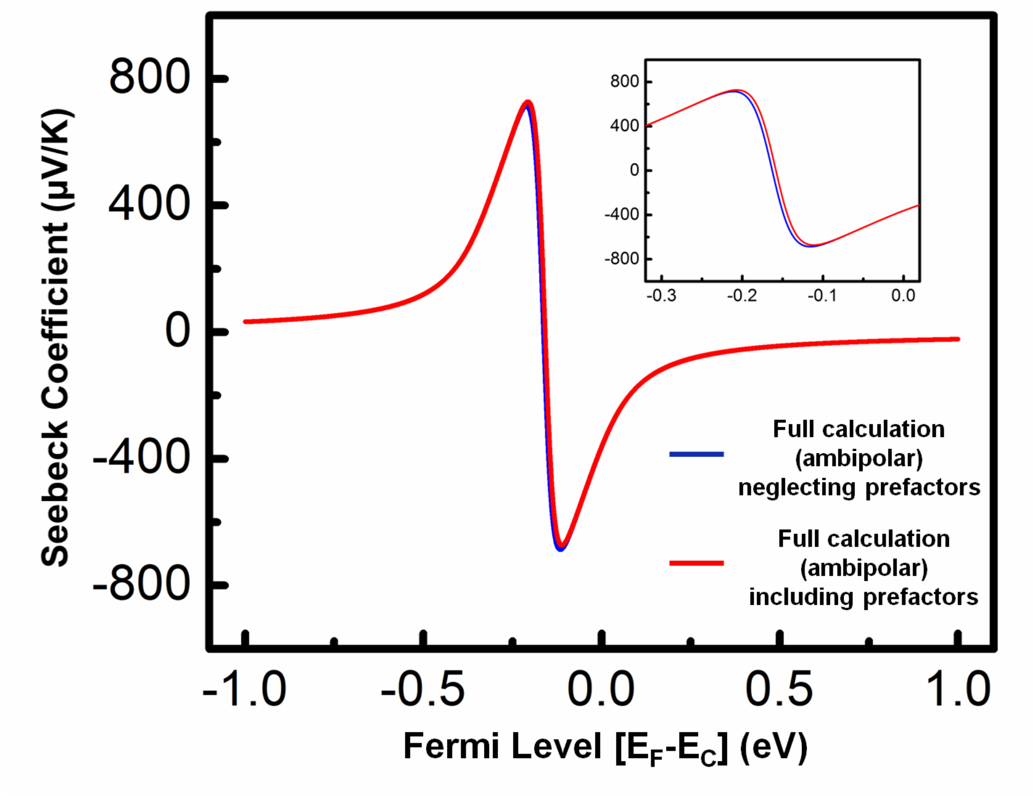

Supplementary Figure 23. **Model depicting the variation in Seebeck coefficient as a function of the Fermi level (E_F) in Te**. The model takes into account both conduction (with effective mass $m_e^* = 0.06 m_0$, where m_0 is the mass of free electron) and valence bands ($m_e^* = 0.114 m_0$) at room temperature. The red and blue curves denote the analytical expression taking into account and neglecting the contribution of prefactors respectively. The inset shows the zoomed-in area of the curve between the conduction band edge (E_C) and the valence band edge.

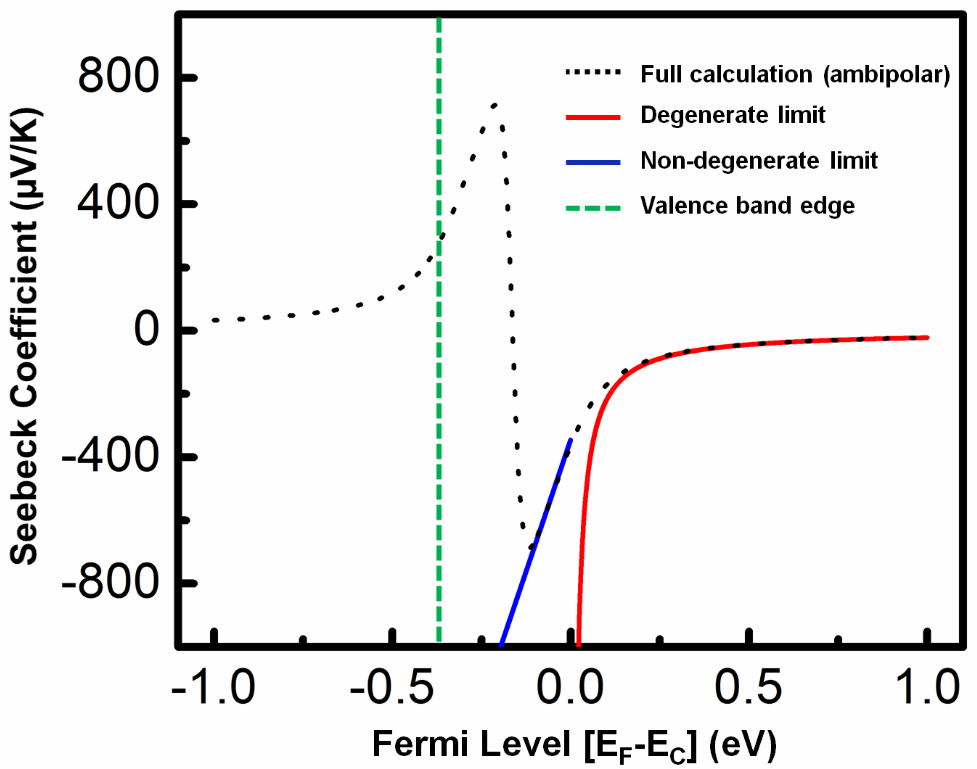

Supplementary Figure 24. **Model depicting the variation in Seebeck coefficient (dotted black curve) as a function of the Fermi level (E_F) in Te in various limits.** The model takes into account both conduction (with effective mass $m_e^* = 0.06m_0$, where m_0 is the mass of free electron) and valence bands ($m_e^* = 0.114m_0$) at room temperature. The red curve denotes the simplified analytical expression for when Te is degenerately doped *n*-type or the degenerate limit, while the blue curve depicts a similar result for the non-degenerate limit but still unipolar doping. At the extreme case of degenerate doping the red curve merges with the dotted black line, while in and around the band edge with non-degenerate doping, the black line matches well with the blue curve. When the effect of the valence band is also taken into consideration for ambipolar transport, the black line deviates from the blue line when the Fermi level is sufficiently far from the conduction band edge (E_C) and near the middle of the band gap.

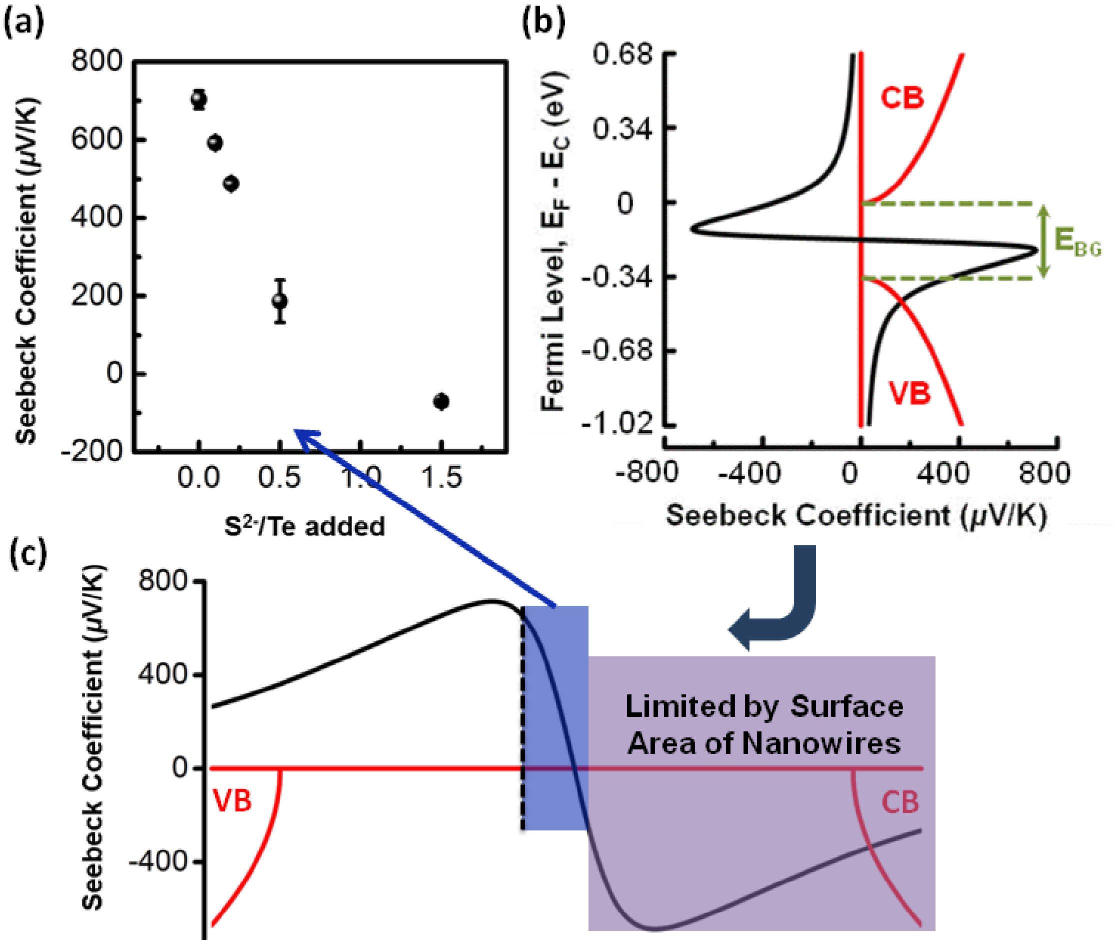

Supplementary Figure 25. **Experimental data and simulated values for variation in Seebeck coefficient by Fermi level tuning.** (**a**) Seebeck coefficient from a series of doped Te nanowire samples versus the amount of S^{2-} added to the exchange solution, normalized to the total number of Te atoms present on the surface of the nanowire. This data is obtained from a different sample batch than Fig. 4a of the main text with ~10-nm diameter Te nanowires. Error bars represent the standard deviation from Seebeck coefficient measurements for each sample and atleast 3 samples for each doping concentration. (**b**) Model depicting the variation in Seebeck coefficient (black curve) as a function of the position of the Fermi level (E_F) in Te. The red curve represents the parabolic band structure used in modeling, with CB and VB referring to the conduction band and the valence band respectively, with E_{BG} (= 0.335 eV) as the band gap and E_C refers to the conduction band edge of bulk Te. (**c**) Zoomed-in area of (b) around the band gap, rotated by 90° so the Fermi level is the x-axis. The shaded area in blue is the region depicted in (a) with the dotted black line denoting the Fermi level for undoped Te nanowires. The Fermi level can be shifted by gradual doping with S^{2-}. The surface area of the nanowires limits the maximum number of dopants that can be added and hence the region in purple depicts the inaccessible region of the band structure of Te.

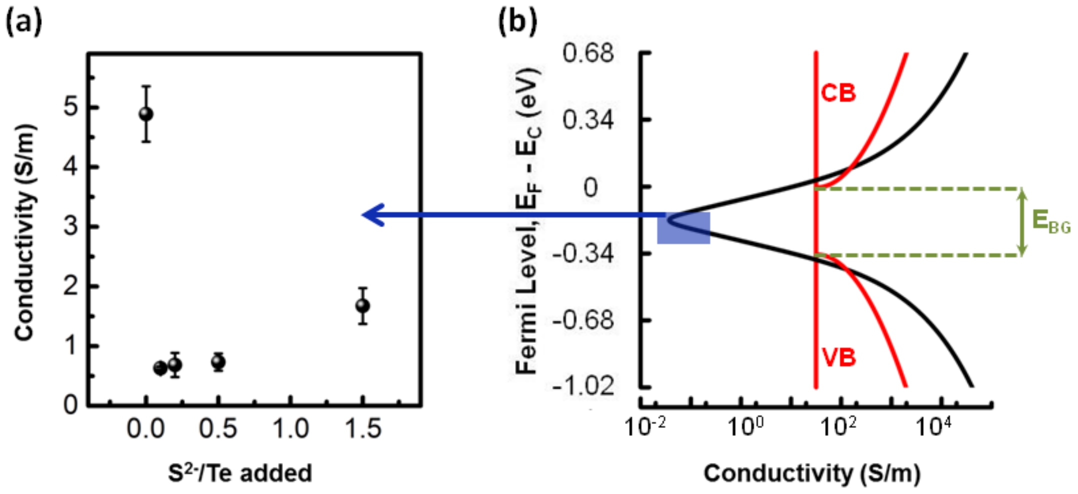

Supplementary Figure 26. Experimental data and simulated values for variation in electrical conductivity by Fermi level tuning. (**a**) Electrical conductivity from the same series of doped Te nanowire samples as shown in Figure S23 versus the amount of S^{2-} added to the exchange solution, normalized to the total number of Te atoms present on the surface of the nanowire. Error bars represent the standard deviation from electrical conductivity measurements for each sample and at least 3 samples for each doping concentration. We observe a decrease in the conductivity at low doping levels and then a steady increase with higher doping amounts which corresponds with our hypothesis that the Fermi level shifts from a level close to the valence band towards the conduction band with doping. (**b**) Model depicting the variation in conductivity (black curve) as a function of the position of the Fermi level in tellurium. The overlaid red curve represents the parabolic band structure used in modeling, with CB and VB referring to the conduction band and the valence band respectively, with E_{BG} (= 0.335 eV) as the band gap and E_C refers to the conduction band edge of bulk Te. The shaded area in blue is the region depicted in (a).

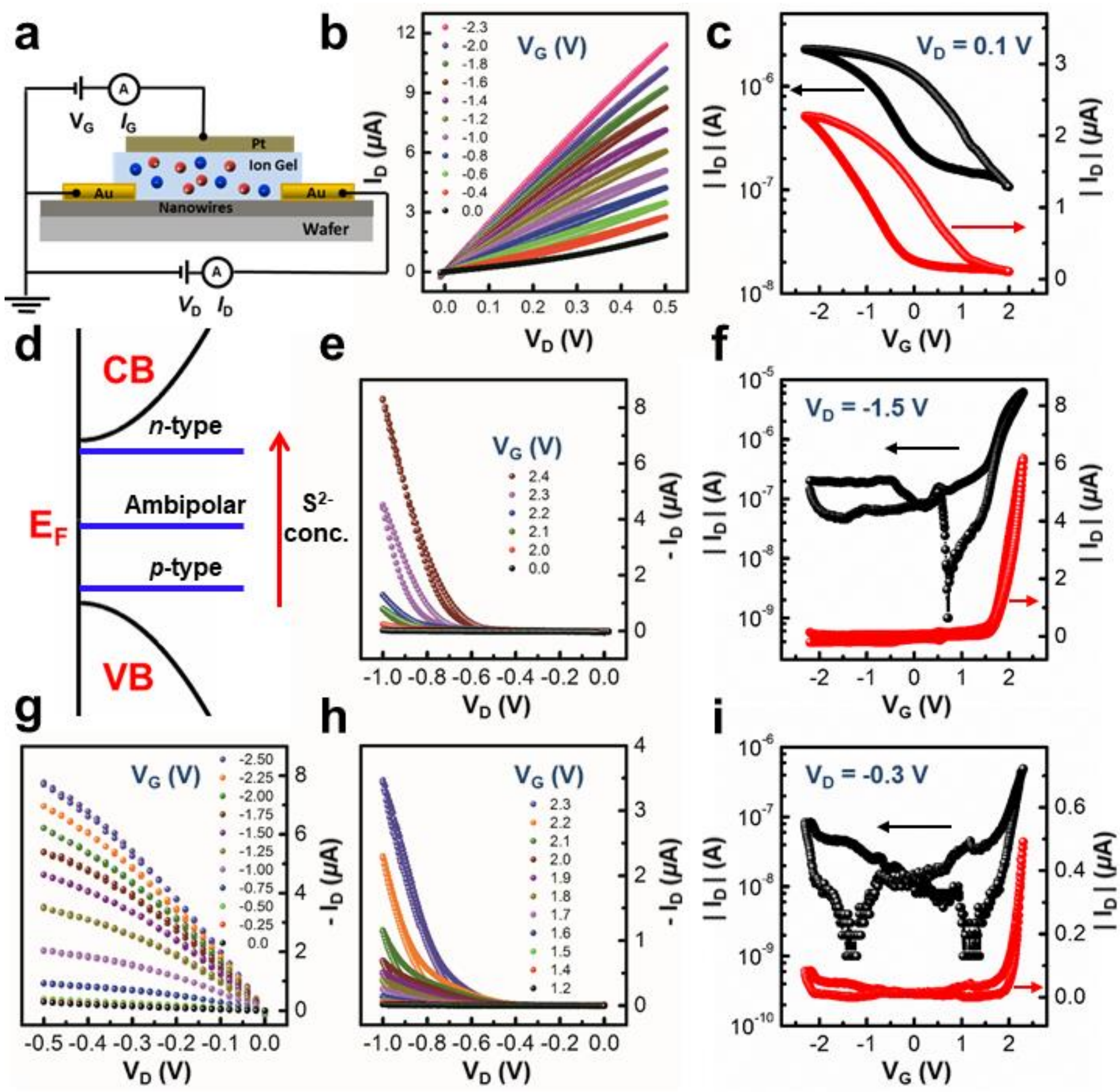

Supplementary Figure 27. Fermi level shifts with doping. **a**, Schematic cross-section (not to scale) of ion-gel-gated thin-film transistors used to characterize the electrical properties of the doped NWs. The length and width of the channel were 100 μm and 2 mm, respectively. Red and blue circles represent positive and negative ions, respectively. **b**, Output characteristics showing drain current, I_D, versus the drain voltage, V_D, for undoped Te NWs at various gate voltages (V_G). **c**, Transfer characteristics for the undoped NW sample in **b** with V_D = 0.1 V. Black and red curves plot the characteristics on logarithm and linear scales respectively. **d**, Energy level diagram depicting the relationship between the location of the Fermi energy (E_F), band-edges, doping concentration and corresponding nature of charge carriers. CB and VB refer to the conduction band and the valence band of Te respectively. **e, f**, Output and transfer characteristics (with V_D = -1.5 V) for heavily-doped Te NWs (~2.4% atomic concentration of sulfur) showing *n*-type transport **g, h, i**, Output and transfer characteristics (with V_D = -0.3 V) for intermediate-doped Te NWs (~1.5% atomic concentration of sulfur) showing ambipolar transport.

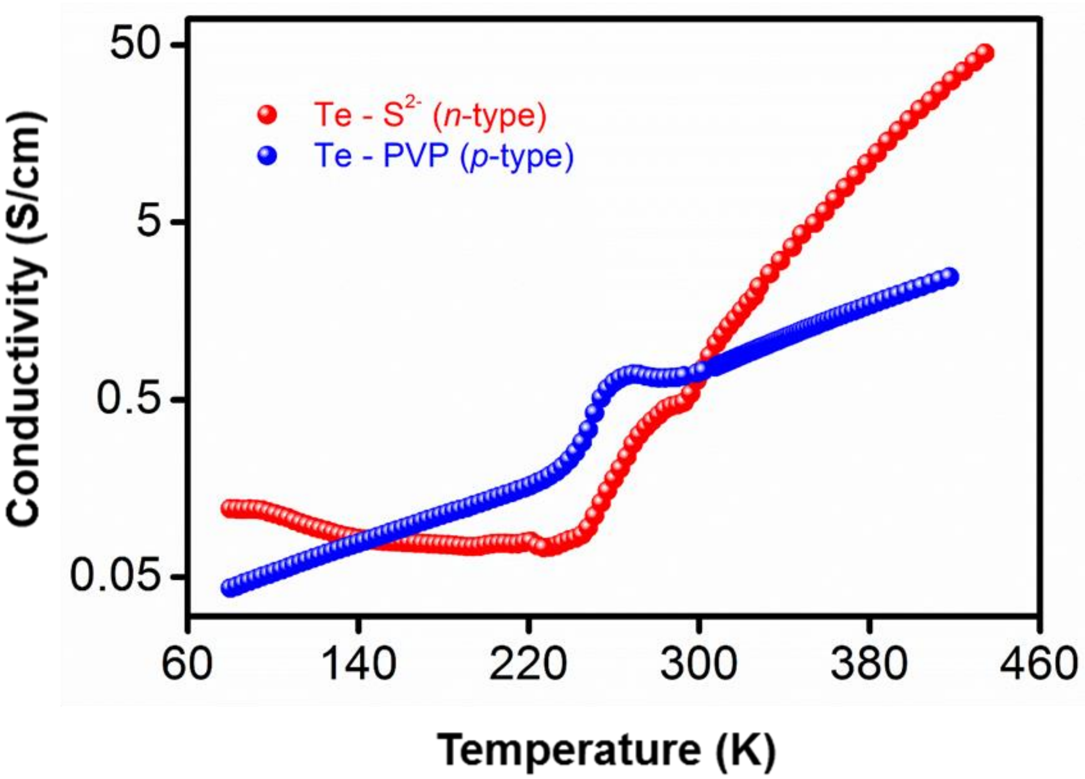

Supplementary Figure 28. Temperature dependent conductivity measurements. Values are shown for thin films of *p*-type PVP-capped and *n*-type sulfur-doped tellurium nanowires. Conductivity values are nearly 0.5 S/cm at room temperature for both samples. For the *n*-type sample, the values rise much more rapidly with increasing temperature as compared to the *p*-type sample and reach ~ 50 S/cm at 155 °C.

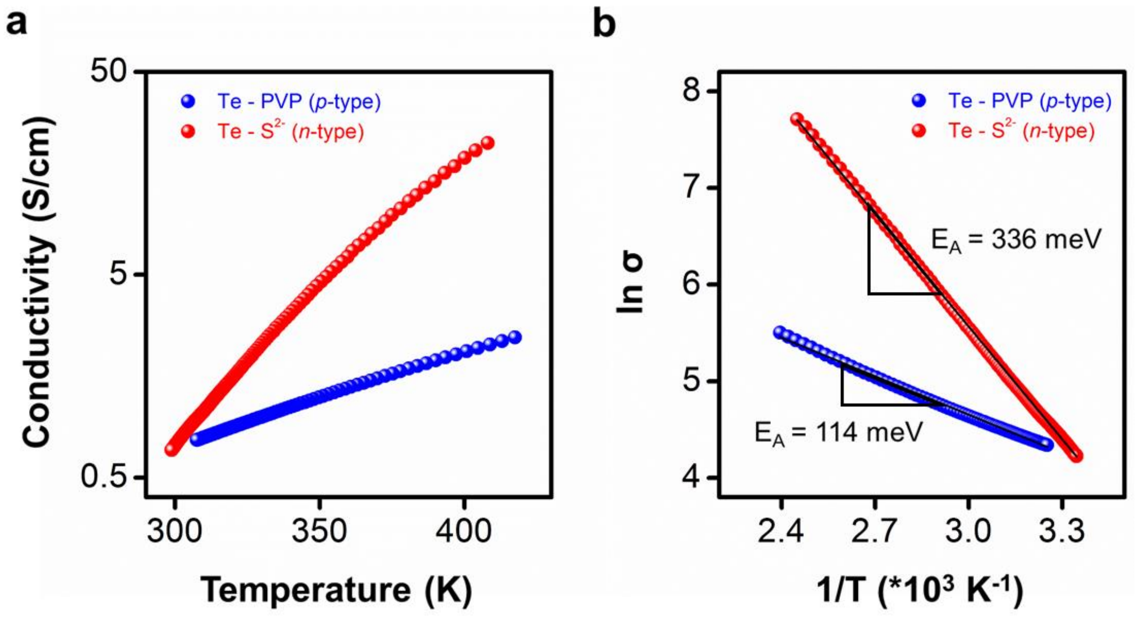

Supplementary Figure 29. Activation barriers to carrier transport using temperature dependent conductivity measurements. (a) Temperature dependent conductivity values plotted on a logarithm scale comparing p-type undoped tellurium nanowires capped with polyvinylpyrrolidone (PVP) and n-type sulfur-doped tellurium nanowires **(b)** Log conductivity (σ) versus inverse temperature (1/T) to calculate the activation energies (E_A) for charge transport in films cast of *p*-type and *n*-type tellurium. The R^2 fit values for the *p*-type and the *n*-type systems were 0.9979 and 0.9997 respectively.

Supplementary Tables

	Dopant (safe/non-toxic/non-hazardous)	Ease of Process (Time/Energy Consumption)	Stability to Environment (Air-stability, Water-stability)	Stability over Time	Solution Processability Post Treatment	Material Identity (Shape, Size) Post Treatment	Precise Control over Doping	Scalability
Talapin, Wang	✘	✓	✘	✘	✘	✓	✘	✘
Sargent	✘	✘	✘	**NA**	✘	✓	✘	✓
Kanatzidis	✘	✘	✘	**NA**	✘	✘	✘	✘
Our Work	✓	✓	✓	✓	✓	✓	✓	✓

Supplementary Table 1: Summary of various nanostructured surface doping strategies and their comparison to our technique. NA refers to properties that were not reported by the authors of those manuscripts.

	Surface dopant	Energy/Time Intensive Treatments	Stability to Environment (air-stable)	Stability over Time	Toxicity Safety	Solution Processability Post treatment	Material Identity (shape,size) Post Treatment	Control over Surface Doping	Scalability
Talapin, Wang	Hydrazine	Few hours	All treatments in glovebox	Hydrazine slowly desorbs over time	Extremely toxic (hydrazine)	Loses colloidal stability	Retains morphology	Either undoped or fully doped	Hazardous hydrazine
Sargent	$CdCl_2$, $CdBr_2$, mercaptopropionic acid, mercaptobutyric acid,	Time consuming – pre treatment with oleylamine for 3 days to replace native oleic ligands and then treatment with final dopants	All treatments in glovebox	Not reported	Uses cadmium-based compounds	Loses colloidal stability	Retains morphology but forms cadmium halide shell	Some control	Yes
Kanatzidis	HCl	Highly energy intensive	All treatments in glovebox	Not reported	Not safe when scaled up	Loses colloidal stability	Loses morphology	Some control	Issues with HCl
Our Work	Na_2S, NaHS, $(NH_4)_2S$, K_2S, KHS	Few hours	Treatment in water in ambient conditions	Extremely stable	Safe	Retains stability	Retains morphology	Fully controlled	Yes

Supplementary Table 2: Details of various nanostructured surface doping strategies and their comparison to our technique. This table provides full detail on the overview given in Supplementary Table 1.

Supplementary Notes

Supplementary Note 1. Colloidal Stability using Zeta Potential Measurements

In the fully exchanged nanowires, the observation that the nanowires do not aggregate and are still dispersible in polar solutions in the absence of any polymer suggests that there exists sufficient electrostatic repulsion between adjacent nanowires to stabilize the colloidal dispersion. In order to confirm this, we perform Zeta potential measurements to establish the charge on the Te-surface for a range of different sulfur concentrations. The binding of the negatively charged S^{2-} ions resulted in a negative ζ-potential in all cases as expected.

Supplementary Note 2. Band structure using density functional theory calculations – origin of n-type transport

The crystal structure of tellurium (Te) contains two types of bonds which have distinct bond lengths, one is ~2.9Å and another is ~3.4Å. As shown in Supplementary Figure 12, the short bonds fasten the Te atoms together to form an atomic chain coiled up with 3-fold symmetry in (001) direction, whereas the longer bonds bind the atom chains together by van-der-Waals interactions to form the Te crystal structures in space. To understand the bonding mechanism more fully, first, we elucidate the origin of the hexagonal shape of pure Te nanowires by calculating the energies of the three prominent surfaces namely (010), (110), and (001), denoted as A, B, and C respectively. The surface energy calculation can be formulized as $E_s = (E_{slab} - nE_{bulk})/2S$. Here, E_s denotes the surface energy, E_{slab} is the total free energy of the slab supercell, n is the number of atoms in the supercell, E_{bulk} is the energy of a Te atom in bulk form, and S is the surface area of the surface of the slab on each side. From our calculations, the surface energies are 18 meV/Å^2, 21 meV/Å^2, and 36 meV/Å^2 respectively for the A, B and C surfaces. During the growth process, the nanowire tries to minimize the total configuration energy, so that surface A (010) is always preferred, leading to the hexagonal shape of the nanowire. In all further calculations, we shall focus our analysis only on the (010) surface, since it is the most stable and exposed surface to the S-adatoms. The nanorod grows along (001) direction with the inter-atomic-plane spacing of 5.93Å.[6] The surface C has much higher energy amongst all three surfaces, proving that the ~2.9Å bond is much stronger than that of ~3.4Å bond.

Allotropes of sulfur occur in various polymorphs with a complex phase diagram due to co-existence of weak S-S VdW bonds and relatively stronger S-S bonds with flexible bond geometries. On the other hand, the cohesive energy of Te-Te is weak (small surface energy proves this). Hence, the surface-adsorbed sulfur on tellurium blends the nature of tellurium and sulfur bonding properties and can thus exhibit a variety of surface/interface atomistic geometries in reality. The two extreme cases of the sulfur-surface adsorptions, therefore, can be categorized as chemical-adsorption (or chemisorption) and physical-adsorption (or physisorption) with almost identical formation energy (difference of nearly 60 meV between chemisorbed and physisorbed S). At around room temperature, due to perturbations from thermal energy, in all likelihood, the real physical system might manifest as an intermediate case incorporating both chemically and physically adsorbed

sulfur. S adatoms can choose to form bond with either other S-atoms or with Te atoms. In chemical adsorption, S-Te bonding dominates over S-S bonding, while in physical adsorption S-S bonding is more pronounced. S-S chains in the latter case enable conductive surface-states as shown in the corresponding band structure for physical adsorption.

The atom-projected band structure reveals conductive surface states that originate from the S-S chains along the surface in the physical adsorbed scenario due to the sulfur band crossing the Fermi level (Supplementary Figure 15). Hence, the charge mobility should be increased greatly around the Fermi level. While, the chemical adsorbed structure does not have the conductive band crossing the Fermi level, it does introduce a new dopant band close to the conduction band edge which can explain the surprising *n*-type behavior of the sulfur-doped tellurium nanowires (Supplementary Figure 16). If we compare the calculated Seebeck coefficients from the DOS for the three different cases cited above – undoped Te and sulfur-doped Te with sulfur adsorbed either physically or chemically (Supplementary Figure 17), we can observe very distinct behavior for the variation in Seebeck coefficient as a function of the Fermi level in each of the three scenarios. In the case where sulfur is physically adsorbed on the surface of tellurium, the S-S chains introduce a lot of surface states which result in switching to negative Seebeck coefficients by shifting the Fermi level to about 25 meV above the valence band (VB) edge of tellurium. Similarly, for the chemically adsorbed case, a Fermi level that is about 190 meV above the VB edge results in negative Seebeck coefficients as compared to 240 meV for undoped tellurium. What these results point out is that, if we assume that the Fermi level in the three different scenarios does not change (*i.e.* no extra charge carriers are added to the system by doping), then with a fixed Fermi level, it is possible to obtain *n*-type doping or *n*-type transport (negative Seebeck coefficients) simply by modifying the local band structure around the Fermi level. For example, if we pin the Fermi level for all three systems at say 230 meV above the VB edge, while we would obtain a Seebeck coefficient of 440 μV/K for the undoped Te (*p*-type), we would obtain Seebeck coefficients of -76 μV/K and -680 μV/K (*n*-type) for the physi-sorbed and chemi-sorbed cases respectively. As discussed before, the two extreme cases of the sulfur-surface adsorptions, categorized as chemical-adsorption (or chemisorption) and physical-adsorption (or physisorption) exist with almost identical formation energy. At around room temperature, due to perturbations from thermal energy, in all likelihood, the real physical system might manifest as an intermediate case incorporating both chemically and physically adsorbed sulfur and thus, the Seebeck coefficients that we observe would be some intermediate value.

Traditional electronic doping strategies employ aliovalent atoms, *i.e.* they use an impurity atom with a different valence compared to the host (*e.g.* boron or phosphorus in silicon) in order to introduce extra charge carriers. A more recent approach to improve the electronic properties of thermoelectrics has been to manipulate the local density of states (resonant levels) in around the Fermi level of the host material (*e.g.* Tl in PbTe, Heremans *et al.* Science 321, 554, 2008, and Sn in Bi_2Te_3, Jaworski and Heremans *et al.* Phys. Rev. B, 80, 233201, 2009). To the best of our knowledge there exists only one other report from Jin and Heremans *et al.*, (Energy Environ. Sci. 2015) that reports iso-electronic doping in materials (indium and gallium in bismuth). Our approach also uses iso-electronic doping (sulfur in tellurium) to modify the band structure and local density

of states in tellurium and introduces an impurity band close to the conduction band of tellurium.

Supplementary Note 3. Energy Dispersive Spectroscopy and X-Ray Photoelectron Spectroscopy.

Since the average diameter of our Te nanowires is nearly 80-nm, only about 2.5% of the total Te atoms constitute the surface. Assuming 100% coverage of the surface Te atoms with S^{2-} atoms would give us only about 2.5% S-species in the samples. While it remains a challenge to accurately quantify the S^{2-} incorporation at low concentrations, qualitatively we are able to use electron dispersive spectroscopy (EDS) and X-ray photoelectron spectroscopy (XPS) to observe a general increase in sulfur concentration with increasing dopant addition. For fully surface-exchanged samples, while quantifying by EDS gives us nearly 2.4% sulfur, quantification by XPS gives us nearly 2.2% sulfur. These values are pretty close to what one would expect with complete surface exchange (2.5%).

Supplementary Note 4. Seebeck Coefficient Calculations.

The Seebeck coefficient is analyzed using a solution to the Boltzmann transport equation (BTE) in the relaxation time approximation, approximating the valence band and conduction band each as a single parabolic band with different effective masses m^*.[7] According to the BTE solution, the Seebeck coefficient S, considering contributions of both electrons and holes, is expressed as[8]

$$S = \frac{2e}{(2\pi)^3 \sigma} \int \tau \vec{v}_k \vec{v}_k \frac{E - E_f}{T} \left(-\frac{df^0}{dE} \right) d^3k \tag{1}$$

where the integral runs over both bands with reference level at the conduction band edge ($E_c = 0$), k is the wavevector, f^0 is the equilibrium Fermi-Dirac distribution function, $\vec{v}_k$ is the group velocity, E_f is the Fermi energy, and σ is the electrical conductivity

$$\sigma = \frac{2e^2}{(2\pi)^3} \int \tau \vec{v}_k \vec{v}_k \left(-\frac{df^0}{dE} \right) d^3k \, . \tag{2}$$

Assuming a single scattering mechanism,[7] the relaxation time τ of electrons or holes can be expressed as a power law with respect to energy[9]

$$\tau \propto \left(E - E_{ref} \right)^{\gamma} \tag{3}$$

where E_{ref} lies at the conduction band minimum for electrons and valance band maximum for holes respectively. γ is a constant determined by the scattering mechanism. Assuming scattering by impurities/vacancies as the dominant scattering mechanism in our samples, γ = 3/2 is used for subsequent analyses.[9] The prefactor used to determine

relaxation time in Supplementary Equation 3 would be canceled out in Supplementary Equation 1 if one carrier type dominated transport. However, in the case of ambipolar transport, since prefactors of electron and holes are not the same, thus they cannot be canceled and hence we need to consider the effect of the prefactor in this regime. The difference in prefactors between electron and hole transport is majorly determined by their respective effective masses.[10] Thus, the relaxation time for each carrier type can be represented as

$$\tau \propto \sqrt{m^*}\left(E - E_{ref}\right)^{\gamma} \qquad (4)$$

where m^* is the effective mass of the carrier. The difference between the constant of proportionalities contributing to the prefactor of electron and hole relaxation times after considering the effective masses are negligible.[10] The comparison between the respective Seebeck coefficients with and without prefactors is shown in Supplementary Figure 23. It can be observed that only in the region of ambipolar transport close to the middle of the band gap, there exists a minimal contribution of the prefactor.

The Seebeck coefficient of tellurium when it behaves as a unipolar *n*-type semiconductor is also cross-checked by the analytical solutions for non-degenerate and degenerate limits. In the case of non-degenerate limit,[11]

$$S = -\frac{k_B}{e}\left[\left(\gamma + \frac{5}{2}\right) + \frac{|E_f|}{k_B T}\right] \qquad (5)$$

while in the degenerate limit,[11]

$$S = -\frac{k_B}{e}\frac{\pi^2}{3}\left(\gamma + \frac{3}{2}\right)\frac{k_B T}{E_f}, \qquad (6)$$

In both cases, the conduction band edge is taken as the reference energy level and the x-axes are plotted as the difference in energies between the conduction band edge and the Fermi level.

Within their respective realms of applicability, these limits both agree with the numerical result obtained from Supplementary Equation 1 as shown in Supplementary Figure 24.

Supplementary Note 5. Comparison with Colloidal Nanostructured Thermoelectric Reports from Other Groups

While a number of groups have demonstrated doping in nanostructures using surface treatments, **no single method offers the precise level of control (over doping and hence carrier concentrations) and stability (over months) that our technique provides**.[12-14] None of the treatments generate colloidal stable (for cheap roll-to-roll fabrication) as well as conductive samples (for better device performance) post-treatment. In contrast, **our method is unique** since it captures the best of all that each of the above mentioned techniques have to provide and does so in an elegant and simple way. In the tables **(Supplementary Tables 1 and 2)**, we compare our technique with other approaches in literature.

In all these cases, the size of the nanocrystals employed varied from a few nanometers to up to 10-15 nanometers where the surface atoms constitute from 20% (~15-nm PbTe, Kanatzidis, JACS 2015)[13] to up to 40-50% (~2-3-nm PbS, Sargent)[14] of the total atoms. Any change to the surface would therefore intuitively have a dramatic effect on the properties of the resultant material. *Impurity atom concentrations at 20% are generally considered alloys rather than dopants.* Finally, it is accepted that the halogen treatment of lead-based nanocrystals leads to the formation of a lead halide layer at the surface of the nanocrystals which can thus be imagined as lead chalcogenide core and lead halide shell nanocrystal rather than a halogen-doped lead chalcogenide nanocrystal. In this sense, referring to the Kanatzidis work as "doping" is a misnomer. Additionally, in these surface treatments, either the nanostructure is undoped or the surface is completely exchanged thus providing limited control over the dopant concentration and hence the resultant properties. In our case, the maximum impurity concentration we have is around 2.5% which allows us to mostly preserve the band structure of the host material whilst tuning the properties through the dopant concentration in contrast to obtaining a wholly new compound. *No study to date has shown such precise control over the electronic properties in colloidal nanomaterials while retaining their nanocrystalline structure.*

Most colloidal nanostructured TE reports that employ surface doping are characterized by following methods to render the materials usable for devices

(a) Heat treatment to 400 °C after the surface treatment to promote insertion of ions into the lattice - thus similar to bulk doping.
(b) Spark plasma sintering – highly energy intensive process.
(c) No longer solution processable after the surface treatment.
(d) Consolidated in a hot press system – not flexible anymore.
(e) After heat treatment and hot press, the particles are sintered (average crystal size deduced from XRD typically increases), which likely results in a large particle size dispersion, thereby detracting from the initial approach of using carefully controlled colloidal syntheses to obtain highly monodisperse nanocrystals that are favorable for the application in consideration. If by using this hot press technique, one obtains sintered polycrystalline and polydispersed nanostructures with little or no control over final grain sizes, one might as well use cheaper techniques like mechanical ball milling.

In contrast, our strategy is different from the earlier works in the following ways:

(a) Our surface treatment is highly controlled and tunable over a wide range (we do not scratch the ends of the spectrum – either heavily doped or completely undoped).
(b) Our surface treatment renders a robust and stable material for months.
(c) Our surface treatment procedure retains the solution processability for flexible electronics.
(d) Our surface treatment preserves the nanocrystalline (size and shape) features of the starting material.
(e) Our surface treatment is cheap and highly scalable for manufacturing.
(f) Our surface treatment is performed in ambient conditions with no heat treatment or additional energy requirements
(g) Our surface treatment employs environmentally benign, recyclable and reusable material (sulfur salts).

Supplementary Methods

Supplementary Method 1. Density Functional Theory Calculations.

To elucidate the hexagonal rod shape formation and the charge transfer effect between S-adatom and the rod, we use the Vienna *ab initio* software package (VASP)[2] to perform the density functional theory calculations, along with the projector augmented-wave (PAW) method[3] to describe the ion-electron interactions and the generalized gradient approximation (GGA)[4] within the Perdew-Burke-Ernzerhof (PBE) framework[5] as the exchange-correlation functional. The surface calculation of tellurium is modeled by a slab supercell with a vacuum thickness of 20 Å to separate the slabs and a tellurium slab thickness of 30Å to prevent the interaction between two termination surfaces. The lattice parameters of the supercell are fixed at its bulk value in the slab in the in-plane direction, and all the atoms are relaxed freely with strict convergence criteria until the forces on each atom are less than 0.01eV/Å and energy difference between ionic relaxation iterations is less than 0.001eV in all the calculations. All calculations use a plane-wave cutoff of 500eV and 21x21x1 mesh for k-space to obtain converged results. Moreover, the magnetic moment is not considered in any of the calculations as tellurium and sulfur are not spin-polarized. The charged slab calculations from periodic super cell algorithm cause issues as the electrostatic energy of such a slab model is conditionally convergent only when infinite vacuum space between surfaces are chosen for calculations, which is non-feasible. Hence, in all the calculations, a neutral system is adopted without introducing excessive charge.

Supplementary References

(1) Hadjichristidis, N.; Pispas, S.; Floudas, G. *Block Copolymers*; John Wiley & Sons: Hoboken, 2003.
(2) Kresse, G.; Furthmuller, J. *Phys. Rev. B* **1996**, *54*, 11169.
(3) Blochl, P. E. *Phys. Rev. B* **1994**, *50*, 17953.
(4) Perdew, J. P.; Chevary, J. A.; Vosko, S. H.; Jackson, K. A.; Pederson, M. R.; Singh, D. J.; Fiolhais, C. *Phys. Rev. B* **1992**, *46*, 6671.
(5) Perdew, J. P.; Burke, K.; Ernzerhof, M. *Phys. Rev. Lett.* **1996**, *77*, 3865.
(6) Zhuge, F.; Yanagida, T.; Fukata, N.; Uchida, K.; Kanai, M.; Nagashima, K.; Meng, G.; He, Y.; Rahong, S.; Li, X.; Kawai, T. *J. Amer. Chem. Soc.* **2014**, *136*, 14100.
(7) May, A. F.; Toberer, E. S.; Saramat, A.; Snyder, G. J. *Phys. Rev. B* **2009**, *80*, 125205.
(8) Cai, J.; Mahan, G. D. *Phys. Rev. B* **2006**, *74*, 075201.
(9) Lundstrom, M. *Fundamentals of carrier transport*; Cambridge University Press, 2009.
(10) Brooks, H. **1955**, 158.
(11) Nolas, G. S.; Sharp, J.; Goldsmid, H. J. *Thermoelectrics: basic principles and new materials developments*; Springer, 2001; Vol. 45.
(12) Wang, R. Y.; Feser, J. P.; Lee, J.-S.; Talapin, D. V.; Segalman, R.; Majumdar, A. *Nano Lett.* **2008**, *8*, 2283.
(13) Ibáñez, M.; Korkosz, R. J.; Luo, Z.; Riba, P.; Cadavid, D.; Ortega, S.; Cabot, A.; Kanatzidis, M. G. *J. Amer. Chem. Soc.* **2015**, *137*, 4046.

(14) Yuan, M.; Zhitomirsky, D.; Adinolfi, V.; Voznyy, O.; Kemp, K. W.; Ning, Z.; Lan, X.; Xu, J.; Kim, J. Y.; Dong, H.; Sargent, E. H. *Adv. Mater.* **2013**, *25*, 5586.
